# Pro‐Vitamin A Biofortified Cavendish Banana: Trait Stability in the Field

**DOI:** 10.1111/pbi.70516

**Published:** 2026-02-03

**Authors:** Jimmy M. Tindamanyire, Jacinta L. Watkins, Cara Mortimer, Bulukani Mlalazi, Jeff Daniells, Rob Harding, James L. Dale, Jean‐Yves Paul

**Affiliations:** ^1^ Centre for Agriculture and the Bioeconomy Queensland University of Technology Brisbane Queensland Australia; ^2^ National Agricultural Research Laboratories National Agricultural Research Organisation Kampala Uganda; ^3^ Agri‐Science Queensland, Department of Primary Industries South Johnstone Queensland Australia

**Keywords:** banana, biofortification, carotenoids, cavendish, genetic engineering, pro‐vitamin a, staple food crop, Uganda, vitamin a deficiency

## Abstract

Vitamin A deficiency (VAD), a major global health concern, has driven efforts to develop staple crops with enhanced pro‐vitamin A (pVA) content. Delivering meaningful nutritional benefits, however, requires technologies that maintain elevated carotenoid levels under field conditions. Previous proof‐of‐concept work demonstrated that pVA content can be substantially increased in Cavendish bananas through genetic modification, providing a platform for transferring the technology into East African Highland banana (EAHB) cultivars relevant to reducing VAD in Uganda. To evaluate performance under agronomic conditions, we conducted multi‐generational field assessments of 27 transgenic Cavendish lines generated from seven constructs enabling constitutive or fruit‐preferred expression of three carotenoid biosynthesis genes: *ZmPsy1*, *MtPsy2a* and *PaCrtI*. Constitutive expression was driven by the maize Ubi promoter, while fruit expression was regulated by Exp1 or ACO promoters. Agronomic performance and fruit carotenoid levels were analysed across three generations to explore factors influencing pVA enhancement. All transgenic lines exhibited increased fruit pVA, with the highest accumulation observed in lines constitutively expressing *MtPsy2a*. Promoter‐transgene combinations significantly affected carotenoid accumulation and the stability of the trait in the field. PVA accumulation was the highest in the initial sucker crop and declined in subsequent ratoons, reflecting sensitivity to seasonal conditions. While ACO‐ and Ubi‐driven lines were less affected by seasonal temperature changes, these variations significantly constrained pVA accumulation in wild‐type and Exp1‐regulated lines. This comprehensive assessment helps elucidate the complex interplay of promoter, isoform, and environmental factors that are essential for the long‐term viability of nutritional interventions aimed at combating VAD in the region.

## 1 Introduction

Vitamin A is an essential micronutrient required for normal vision, immune function, cellular communication, and reproduction (Tanumihardjo et al. 2016). It can be obtained directly from animal‐derived foods such as liver, eggs, and dairy products, or synthesised in the human body from pro‐vitamin A (pVA) carotenoids, including α‐ and β‐carotene, which are found in plant‐based foods (Menezes and Almeida 2024; Office of Dietary Supplements 2025).

Vitamin A deficiency (VAD), defined as serum retinol below 0.70 μmol/L, is a major global public health concern, particularly in low‐ and middle‐income countries (Menezes and Almeida 2024). VAD increases the risk of infections and related complications, contributes to eye diseases such as xerophthalmia and night blindness, impairs growth, and is associated with adverse maternal outcomes. It is a leading cause of preventable childhood blindness and contributes significantly to child mortality due to diarrhoeal diseases, measles, and respiratory infections (Wiseman et al. 2017). Globally, VAD has been estimated to cause approximately 2.5 million preventable deaths each year, with the highest burden in sub‐Saharan Africa (Ssentongo et al. 2020).

In 2005, the World Health Organization (WHO) estimated that 33.3% of preschool‐aged children (under five years) were affected by VAD, with the highest burdens in South‐East Asia and Africa (World Health Organization 2009). Although global prevalence declined slightly to 29% by 2015, sub‐Saharan Africa (48%) and South Asia (44%) continued to exhibit disproportionately high rates (Stevens et al. 2015). More recent assessments show that VAD still affects nearly 30% of children and adolescents in low sociodemographic index regions, more than twice the global average of 14.7% (Song et al. 2023). Children under five remain especially vulnerable, with prevalence approaching 20% globally and reaching 30.6% in sub‐Saharan Africa. In Uganda, national survey data indicate a decline in VAD prevalence from 27.9% in 2001 to 15.1% in 2016 (Uganda Bureau of Statistics 2012, 2018), and more recent estimates suggest further reductions to 8.3% in urban areas and 9.0% in rural populations (Ssentongo et al. 2020; Uganda Bureau of Statistics 2023). Although interventions such as high‐dose supplementation, dietary diversification, food fortification and the biofortification of some staple crops have contributed substantially to national gains (Tanumihardjo and Furr 2013; Keats et al. 2019), a large proportion of the rural poor continue to benefit only marginally, underscoring the need for accessible, food‐based solutions that reach vulnerable communities.

Although supplementation has been shown to reduce all‐cause child mortality by up to 24% when effectively implemented (Ssentongo et al. 2020), its coverage remains inconsistent especially across regions with the highest burden of child mortality. The continued high prevalence of VAD in Uganda and across sub‐Saharan Africa points to critical gaps in implementation, sustainability, and equity of current strategies (Mkambula et al. 2020; Victora et al. 2021), and highlights the need for more durable, context‐specific solutions. Biofortification of staple crops is a sustainable, long‐term strategy to help alleviate VAD by enhancing the nutrient content of widely consumed foods. It can be achieved through agronomic interventions, conventional breeding, genetic modification, and, more recently, gene editing technologies (Cakmak 2008; Paul et al. 2017; Hossain et al. 2019; Kumar et al. 2022). Orange‐fleshed sweet potato, rich in beta‐carotene, has demonstrated efficacy in improving vitamin A status amongst children in sub‐Saharan Africa through numerous controlled trials and large‐scale adoption, with over 6.8 million households currently cultivating the crop in Africa and South Asia (Low and Thiele 2020; Ahoudou et al. 2025). Similarly, pVA‐biofortified maize varieties offer a complementary approach in regions where maize is a dietary staple (Bai et al. 2011; Gannon et al. 2014; Baudron et al. 2024).

The East African Highland Banana (EAHB, AAA Group, Mutika/Lujugira Subgroup) is the primary staple food in the Great Lakes region of East Africa, particularly in Uganda, where it is consumed in larger quantities than most other carbohydrate‐rich crops. Despite its dietary importance, commonly cultivated EAHB varieties are low in pVA carotenoids, providing only around 25% of the estimated average requirement (EAR) for vitamin A (Mbabazi et al. 2020). This shortfall contributes to the high incidence of VAD in banana‐dependent populations. However, the crop's widespread consumption presents a strategic and valuable opportunity for improving vitamin A intake through targeted biofortification initiatives.

One such initiative is the Banana21 project. This collaborative project set out to increase to 50% the contribution of EAHBs to the vitamin A EAR by engineering and delivering pVA‐enriched EAHBs to Ugandan populations (Paul et al. 2017). Over more than a decade, collaborative teams in Uganda and Australia have worked at improving the genetic modification of various banana cultivars and sourcing the right combination of transgenes and regulatory elements to achieve sustainable high levels of fruit pVA in banana.

The initial “proof‐of‐concept” for the technology was established in Australia, where 244 independent transgenic Cavendish banana lines were analysed in the field. This first field trial (FT‐1) was crucial in demonstrating the feasibility and effectiveness of the approach, showing that expression of a single *phytoene synthase* (*psy*) transgene was sufficient to induce significant accumulation of pVA carotenoids in banana fruit without compromising yield (Paul et al. 2017). While technical expertise and transformation capacity were progressively transferred to the National Agricultural Research Organisation (NARO) of Uganda for implementation in preferred EAHB cultivars, parallel efforts in Australia focused on characterising, refining, and optimising the strategy. Although FT‐1 provided critical information, every line tested was only represented by a single biological replicate in order to maximise the number of independent lines tested. To address the limitations of this approach and validate trait stability and agronomic performance, a second field trial (FT‐2) was subsequently conducted. This follow‐up trial assessed a subset of 30 selected independent lines each represented by ten suckers (field‐derived clonal replicates).

Cavendish provides a robust, well‐characterised genetic background in which promoter performance, isoform behaviour, multi‐generational stability, and environmental responsiveness can be rigorously evaluated. The ultimate deployment target for this biofortification strategy is the East African Highland banana (EAHB), and the knowledge generated from FT‐2 directly informs the design, selection, and optimisation of pVA‐enhanced EAHB lines currently under development and field testing in Uganda (Buah et al. 2025).

This manuscript reports the thorough field assessment of these plants, including phenotypic observations, agronomic characteristics, and fruit pVA performance across three successive generations. It also explores the molecular and biochemical basis of the *ZmPSY1*‐associated ‘golden’ phenotype observed in certain lines and establishes the foundational stability data required to guide future deployment and regulatory advancement of pVA‐enhanced banana in East Africa.

## 2 Results

The transgenic lines reported in this study originated from seven transformation constructs designed specifically for the constitutive or fruit preferred expression of three genes involved in carotenoid biosynthesis (Figure S1). These were, the *psy* transgenes from either maize (*ZmPsy1*) or the Fe′i banana ‘Asupina’ (*MtPsy2a*) and the *phytoene desaturase* gene *PaCrtI* from the soil bacteria *Pantoea ananatis* . Constitutive expression was driven by the maize polyubiquitin (Ubi) promoter while fruit expression was controlled by either the Expansin 1 (Exp1) or ACC oxidase (ACO) promoters. Lines were selected based on (i) comparing the expression of *ZmPsy1* to that of *MtPsy2a* with all three promoters, (ii) availability of suckers for planting FT‐2, (iii) limited space availability and (iv) a particular interest in studying the ‘golden’ tissue phenotypes reported by Paul et al. (2017).

All 30 lines were fully characterised to assess the presence of the respective transgene(s) by PCR analysis (Table S2; Figure S2), transgene expression by RT‐PCR (Figure S3) and transgene copy number by Southern blot analysis (Table S3).

### 2.1 Phenotypic and Agronomic Assessment of Field Grown Transgenic Bananas Across Consecutive Generations

To evaluate the performance of transgenic plants, phenotypic and agronomic characteristics were recorded across three generations: the sucker crop (S) followed by the sucker first ratoon (SR1) and second ratoon (SR2) crops (Table 1, Tables S4–S6). Data from three of the 30 lines planted in FT‐2, FT328, FT493 and FT588 were excluded due to insufficient observations.

**TABLE 1:** Phenotypic observations and main agronomical data recorded from selected pVA‐biofortified ‘Cavendish’ banana lines investigated.

Line ID	Promoter‐*transgene*	Phenotypes				Agronomic characteristics (three crop cycles average)					
		Leaf	Fruit flesh	Fruit peel	Other	Plant height (cm)	Plant cycle time (days)	Bunch cycle time (days)	Bunch weight (kg)	Finger number	Finger length (cm)
FT162	Wild‐type	Green	Cream	Green	NA	167.0	270.4	105.4	25.4	163.1	19.9
FT166		Green	Cream	Green	NA	186.0	287.3	106.4	30.1	183.2	20.9
FT167^a^		Green	Cream	Green	NA	181.9	268.0	109.9	28.7	172.9	20.9
FT430^a^		Green	Cream	Green	NA	191.5	279.7	103.8	32.7	181.2	21.5
FT448		Green	Cream	Green	NA	202.4	279.5	112.0	29.9	187.7	20.9
FT187^a^	Exp1‐*ZmPsy1*	Early golden	Cream	Green	Yellow petiole	182.3	278.5	106.5	29.8	169.1	22.0
FT192^a^		Early golden/orange striped	Yellow	Green	Yellow petiole	138.9*	314.3	181.4*	10.2*	109.9*	16.6*
FT201		Early golden	Cream	Green	Yellow petiole	169.5	283.2	105.2	26.4	158.8	21.0
FT317		Early golden/orange striped	Cream	Green	Yellow petiole	142.5*	317.7	114.7	14.3*	108.7*	17.6*
FT538		Early golden	Cream	Green	NA	183.7	271.0	103.9	29.9	179.3	21.0
FT467^a^	ACO‐*ZmPsy1*	Orange striped	Cream	Golden	Yellow petiole	191.9	283.9	102.1	29.1	173.5	20.6
FT475^a^		Orange striped	Yellow	Golden	Yellow petiole	189.3	297.3	107.8	28.0	181.8	20.2
FT479^a^		Orange striped	Yellow	Golden	Yellow petiole	178.6	394.3*	107.7	21.4*	138.5	19.0
FT483		Orange striped	Yellow	Golden	Yellow petiole	161.3	345.0*	115.5	21.4*	170.0	16.5*
FT584^a^	ACO‐*ZmPsy1* + Exp1‐*PaCrtI*	Orange striped	Yellow	Golden	Yellow petiole	283.7*	245.8	109.3	17.5*	121.9*	20.5
FT585^a^		Orange striped	Cream	Golden	Yellow petiole	188.8	272.9	104.6	30.0	173.2	21.2
FT587		Orange striped	Yellow	Golden	Yellow petiole	264.9*	254.3	106.5	15.1*	119.4*	19.4
FT287^a^	Ubi‐*ZmPsy1*	Green	Deep yellow	Green	NA	166.8	358.4*	119.4	13.0*	160.2	12.9*
FT309^a^		Green	Deep yellow	Green	Small plant	125.0*	542.3*	107.8	4.9*	76.2*	13.8*
FT242	Exp1‐*MtPsy2a*	Green	Cream	Green	NA	174.8	288.5	100.2	24.1	145.5	20.5
FT246^a^		Green	Yellow	Green	Yellow petiole	138.2*	273.9	106.5	11.6*	102.5*	16.0*
FT341^a^		Green	Cream	Green	NA	193.5	268.0	110.5	32.5	189.1	21.3
FT342^a^		Green	Cream	Green	NA	184.1	276.8	108.1	30.8	177.8	21.5
FT497^a^	ACO‐*MtPsy2a*	Green	Yellow	Green	NA	170.0	278.5	106.5	26.6	160.9	20.4
FT504		Green	Yellow	Green	Yellow petiole	165.4	267.4	113.6	21.1*	141.5*	19.5
FT508		Green	Yellow	Green	Yellow petiole	190.6	279.3	109.4	30.2	168.8	21.0
FT511		Green	Yellow	Green	Yellow petiole	187.0	273.4	104.0	31.8	171.4	21.2
FT518^a^		Green	Deep yellow	Green	Yellow petiole	161.5	286.1	106.0	19.9*	149.1	19.7
FT294	Ubi‐*MtPsy2a*	Green	Yellow	Green	Yellow petiole	159.0	282.0	103.7	20.4*	130.0*	19.6
FT295^a^		Green	Cream	Green	NA	187.0	278.0	104.6	28.8	166.0	21.1
FT324^a^		Green	Yellow/Pink	Green	Yellow petiole	150.3*	335.7*	119.8	15.0*	107.3*	18.8*
FT330		Green	Cream	Green	NA	346.9*	263.8	95.7	22.6*	121.8*	20.9

*Note:* Data are average of 4 biological replicates over 3 generations (*n* = 12) except for FT309 for which data could not be recorded in the third generation (*n* = 8) and FT483, 3 biological replicates (*n* = 9).

^a^ Plant line selected for gene expression studies. NA, data not available. Phenotypic observation made on the sucker crop.

* Indicates values that are statistically different from the average of the control wild‐type data at 95% confidence (ANOVA and Tukeys HSD post hoc test).

**FIGURE 1:**
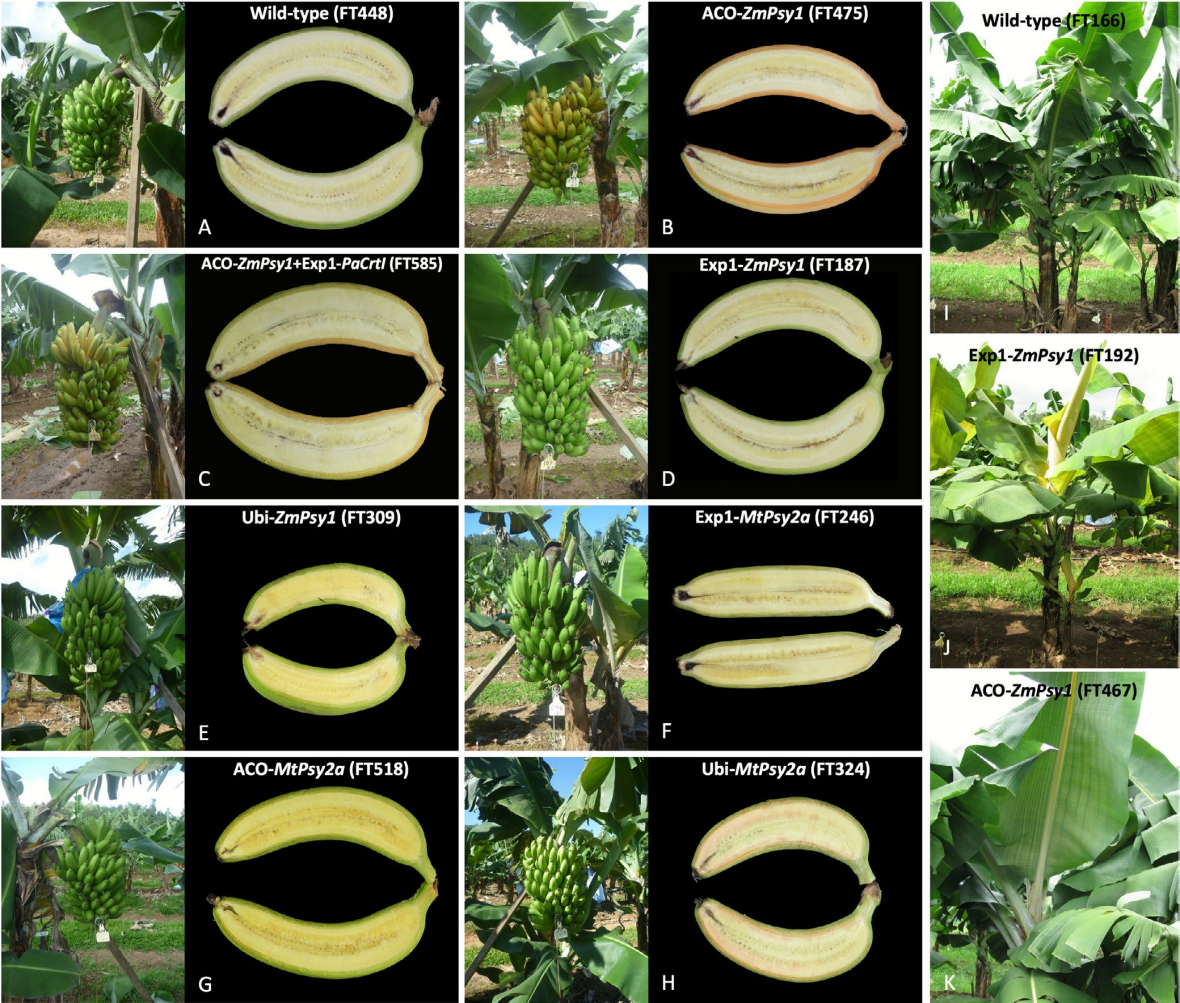
Characteristic bunch and fruit phenotypes from wild‐type and transgenic ‘Cavendish’ banana lines grown in the field. Mature bunches ready to harvest and longitudinal sections of mature green fruit exposing the flesh are shown for wild‐type line FT448 (A); ACO‐*ZmPsy1* line FT475 (B); ACO‐*ZmPsy1* + Exp1‐*PaCrtI* line FT585 (C); Exp1‐*ZmPsy1* line FT187 (D); Ubi‐*ZmPsy1* line FT309 (E); Exp1‐*MtPsy2a* line FT246 (F); ACO‐*MtPsy2a* line FT518 (G); Ubi‐*MtPsy2a* line FT324 (H); wild‐type line FT166 (I); Exp1‐*ZmPsy1* line FT192 (J) and ACO‐*ZmPsy1* line FT467 (K).

All wild‐type (WT) control plants displayed normal development and agronomic characteristics within expectations of commercially grown ‘Dwarf Cavendish’ bananas (Table 1, Figure 1A,I). Across the generations control plants matured to an average height of 186 ± 13 cm with large, expanded green leaves, produced bunches of an average weight of 29.4 ± 2.7 kg within 277 ± 8 days of planting with the fruit presenting a characteristic pale creamy colour (Figure 1A). This contrasts with all transgenic lines expressing *ZmPsy1* under the control of the Exp1 promoter, which in all generations displayed a distinctive ‘golden’ leaf phenotype at the early stages of development (Figure 1J) consistent with observation of these lines in the plant crop in FT‐1 (Paul et al. 2017). The Exp1‐*ZmPsy1* lines also all developed a yellow petiole on newly emerged leaves and in the case of lines FT192 and FT317 displayed orange streaks running perpendicular to venation on the leaf lamina similar to ACO‐*ZmPsy1* line FT467 (Figure 1K). The lines exhibiting the orange streak phenotype on the leaf lamina also showed several agronomic performance impairments across the crop cycles including plants with significantly shorter height (*p* < 0.001) and reduced bunch weights (*p* < 0.001) due to both shorter fingers (*p* < 0.001) and fewer fingers per bunch (*p* < 0.001) compared to the wild‐type (Table 1). However, in lines that exhibited only the ‘golden’ leaf and not the orange striped phenotype (FT187, FT201 and FT538), agronomic performance was comparable to the controls (Table 1). When *ZmPsy1* was under the control of the ACO promoter, either by itself or co‐expressed with the Exp1‐*PaCrtI* construct, the resultant plants also developed the yellow petiole and the orange striped phenotypes. However, in these cases, the orange striped leaf phenotype did not consistently coincide with a reduction to agronomic properties. For example, lines FT467 and FT475 (ACO‐*ZmPsy1*) and FT585 (ACO‐*ZmPsy1* + Exp1‐*PaCrtI*) all performed comparably to the control plants. Interestingly, all lines where *ZmPsy1* was under the control of the ACO promoter, regardless of co‐expression of *PaCrtI*, displayed a ‘golden’ peel phenotype where immature bunches emerged with a bright yellow/orange colour which partially turned green over the course of fruit development (Figure 1B,C). This phenotype was not observed when *ZmPsy1* was regulated by the Exp1 or Ubi promoters (Figure 1D,E), nor when *MtPsy2a* was expressed (Figure 1F–H). When *ZmPsy1* was constitutively expressed under the Ubi promoter, plants developed green ‘true‐to‐type’ leaves whilst producing fruit flesh with an intense yellow colouration (Figure 1E). Both Ubi‐*ZmPsy1* lines investigated in this study, however, generated plants with significantly longer plant cycle times (*p* < 0.001) and lower bunch weights (*p* < 0.001) due to shorter fingers (*p* < 0.001) with line FT309 also developing mature plants with significantly reduced height (*p* < 0.001).

Unlike ACO‐*ZmPsy1* and Exp1‐*ZmPsy1* lines, all lines transformed with the banana gene *MtPsy2a* displayed a ‘true‐to‐type’ phenotype developing green leaves and green fruit peel in immature bunches. When *MtPsy2a* was driven by the Exp1 promoter all lines performed comparably to wild‐type, except for FT246 where mature plants were stunted in height (*p* < 0.001) and developed smaller bunches due both a reduction in the number of fingers per bunch (*p* < 0.001) and a reduction in finger length (*p* < 0.001). When the ACO promoter was used to regulate the expression of *MtPsy2a*, lines generally performed well agronomically, however, two lines (FT504 and FT518) developed reduced bunch weights (*p* < 0.001) with this reduction attributed to a lower finger number in line FT504 (*p* = 0.016). Fruit flesh from these lines were characterised by a deep yellow coloration (Figure 1G). Similar to the Ubi‐*ZmPsy1* lines, constitutive expression of *MtPsy2a* had an impact on plant physiology. Of the four Ubi‐*MtPsy2a* lines observed across multiple generations, only one, FT295, closely resembled the wild‐type with no agronomic differences. In contrast, lines FT294, FT324 and FT330 all produced bunches with significantly lower weight (*p* < 0.001) due to lower number of fingers per bunch (*p* < 0.001). Lines FT324 and FT330 also exhibited alterations in plant height with line FT330 maturing to a significantly taller height than the wild‐type (*p* < 0.001). On the other hand, plants from line FT324 were significantly shorter than controls (*p* < 0.05), had a significantly longer cropping cycle (*p* < 0.05) and consistently produced fruit with a distinctive variegated orange flesh colouration (Figure 1H).

Overall, many PSY over‐expression lines performed comparably to the Dwarf Cavendish controls, however, when alterations to agronomic properties were observed, generally, multiple traits were impacted simultaneously.

### 2.2 Carotenoid Profile and pVA Trait Stability in the Fruit of Wild‐Type and Transgenic Lines Across Successive Generations

**FIGURE 2:**
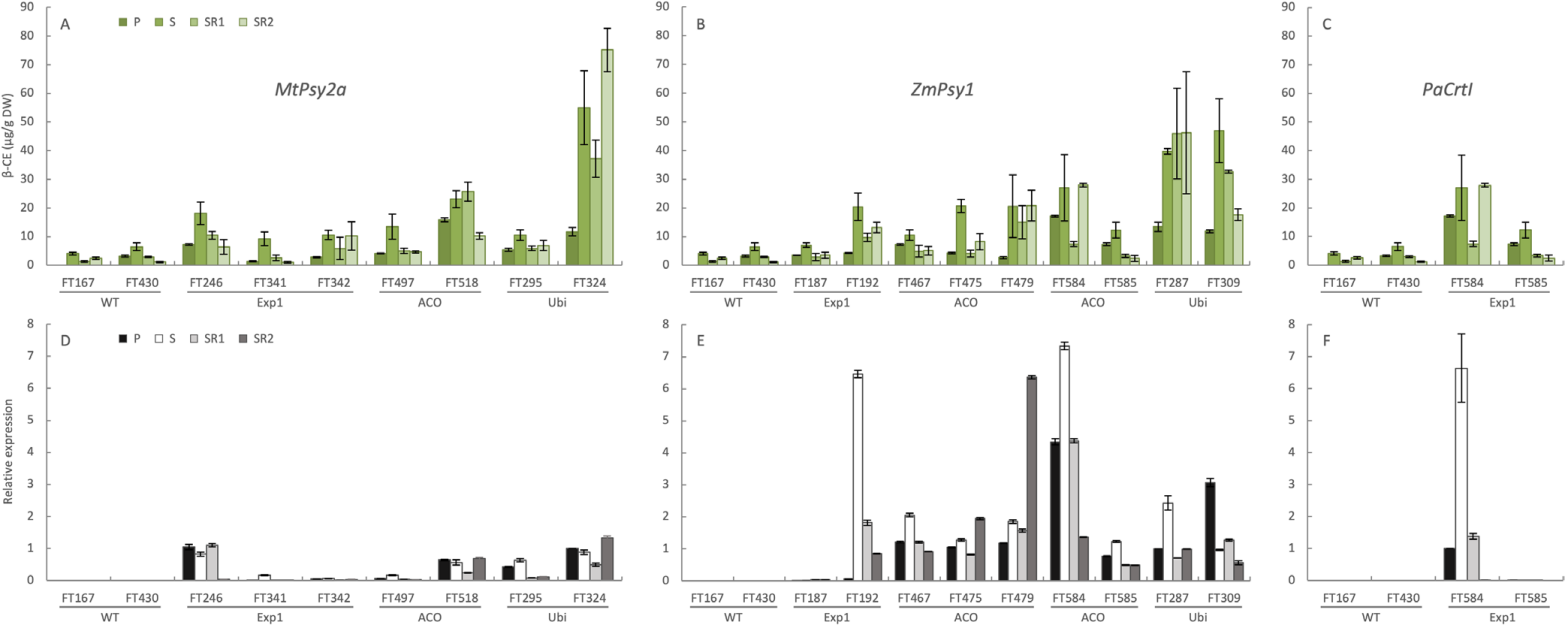
Carotenoid accumulation and transgene mRNA abundance in the fruit of transgenic banana lines. Carotenoid content in the fruit of line harbouring the *MtPsy2a* (A), *ZmPsy1* (B) and *PaCrtI* (C) transgenes were measured by HPLC. Data presented for the PC is an average of 3 technical replicates (*n* = 3) expressed as β‐carotene equivalents (β‐CE) while data presented for the sucker crop (S); sucker ratoon 1 crop (SR1) and sucker ratoon 2 crop (SR2) are an average of 3 technical replicates for 4 biological replicates (*n* = 12). Relative transcript levels from *MtPsy2a* (D), *ZmPsy1* (E) and *PaCrtI* (F) were calculated using the ΔΔCT method, normalised with 2 reference genes and expressed relative to the *MtPsy2a* transcript level in the plant crop (P) of line FT324. Error bars = ±SD. Lines FT167 and FT430 are wild‐type while transgenic lines FT246, FT341 and FT342 (Exp1‐*MtPsy2a*); FT497 and FT518 (ACO‐*MtPsy2a*); FT295 and FT324 (Ubi‐*MtPsy2a*); FT187 and FT192 (Exp1‐*ZmPsy1*); FT467, FT475 and FT479 (ACO‐*ZmPsy1*); FT584 and FT585 (ACO‐*ZmPsy1* + Exp1‐*PaCrtI*) and FT287 and FT309 (Ubi‐*ZmPsy1*).

Stable inheritance of the increased pVA trait is critical for reliable nutritional enhancement and the long‐term viability and deployment of biofortified cultivars. Given the variability previously observed in pVA content between the individual transgenic lines (Paul et al. 2017), carotenoid profiling was conducted across successive vegetative generations to evaluate whether trait performance was consistent over time. Table S7 show the details of the 722 fruit samples collected and analysed by HPLC in this study. Common to all lines including the wild‐type was an increase in pVA, reported as β‐carotene equivalents (β‐CE), in the fruit from the sucker crop (S) compared to the plant crop (P) (Figure 2A–C). The largest increase observed was for ACO‐*ZmPsy1* line FT483 where fruit β‐CE content increased from 1.69 ± 0.26 μg/g DW in the plant crop to 18.8 ± 6.6 μg/g DW in the sucker crop representing an 11‐fold increase (Table S8). Likewise, wild‐type line FT166 showed a 6.7‐fold increase in β‐CE content between the plant and sucker crops, one of the largest increases in accumulation observed across all genotypes in this study. In contrast to the sucker crop, the following generation (SR1) was characterised by a general modest reduction in β‐CE accumulation across most lines with the exception of the transgenic ACO‐*MtPsy2a* line FT518 which displayed around 11% increase in β‐CE content. Only two lines demonstrated consistent β‐CE accumulation across the S, SR1 and SR2 crops: ACO‐*ZmPsy1* line FT479 and Ubi‐*ZmPsy1* line FT287, both of which are characterised by high pVA content. In contrast, all wild‐type lines exhibited high intergenerational variability. For example, control line FT162, declined in β‐CE content from 4.65 ± 0.5 to 1.57 ± 0.3 and finally 0.98 ± 0.1 μg/g DW in the S, SR1 and SR2 crops, respectively. High variability was also observed in some transgenic lines, such as ACO‐*ZmPsy1* line FT475, Exp1‐*MtPsy2a* lines FT246 and FT341, as well as ACO‐*MtPsy2a* line FT518. In the SR2 crop, line FT324, constitutively expressing *MtPsy2a* via the Ubi promoter, recorded the highest average β‐CE content of any line in any cropping season reaching 75.15 μg/g DW. This represents a 39‐fold enhancement compared to Dwarf Cavendish controls harvested in the same generation. This line is the highest overall β‐CE accumulating lines across the generations. Other lines with promoter‐transgene combinations supporting consistently high levels of β‐CE accumulation across all generations included FT287 (Ubi‐*ZmPsy1*), FT479 (ACO‐*ZmPsy1*), and FT518 (ACO‐*MtPsy2a*) which represent 24‐, 11‐ and 5‐fold accumulation compared to the average of the controls at the SR2 generation.

Given that high variation in fruit β‐CE content was observed across generations in most lines, qRT‐PCR was performed to assess whether this variation could be attributed to differences in transgene expression. As expected, transgene expression was undetectable in wild‐type controls (Figure 2D–F). Across all transgenic lines, substantial variation in transgene expression was observed between cropping generations. However, transcript abundance did not consistently correlate with fruit β‐CE content. For example, Ubi‐*ZmPsy1* line FT287, which accumulated amongst the highest β‐CE levels across all lines, exhibited only modest transgene expression. Additionally, in ACO‐*ZmPsy1* line FT479, *ZmPsy1* expression was approximately 4‐fold higher in the SR2 compared to the SR1 crop; however, no significant difference in β‐CE accumulation was detected between these two generations in this line.

Detailed carotenoid profiling was also conducted on mature green fruit across vegetative generations to evaluate the composition of individual carotenoids, including the non‐pVA carotenoid lutein, to determine whether differences between lines reflected underlying variation in metabolic flux. In wild‐type fruit, the proportion of lutein progressively increased across the generations with line FT430 accumulating 46%, 71% and 80% lutein in the S, SR1 and SR2 crops, respectively (Figure S4). The maximum proportion of β‐carotene observed in wild‐type fruit was 13% for the all‐*trans* isomer and 1% for the *cis*‐isomer in crop S for FT430 with similar proportions recorded for the other investigated wild‐type line, FT167. The majority of transgenic lines displayed similar proportions of individual carotenoids compared to controls across the vegetative generations, however, there were two notable exceptions. Ubi‐*MtPsy2a* line FT324 maintained a relatively stable profile and accumulated only 13%–14% lutein but substantially higher proportions of α‐carotene (60%–62%) and β‐carotene (23%–25%) across all generations (Figure S4). Similarly, Ubi‐*ZmPsy1* line FT287 exhibited a reallocation of flux to the β‐branch of the carotenoid biosynthesis pathway accumulating 12%–21% lutein, 56%–61% α‐carotene and 20%–29% β‐carotene across all generations investigated. Both FT287 and FT324 were amongst the top‐performing lines with some of the highest pVA accumulation recorded across all lines investigated in this study.

Although β‐CE content at the mature green stage was prioritised given its relevance to the typical consumption stage of EAHBs, pVA levels were also assessed at the full ripe stage in the transgenic lines. Overall, ripening led to a modest increase in β‐CE content in all wild‐type lines, with an average of a 25% increase observed in the S crop. An exception was noted in the SR1 crop of wild‐type line FT430, where β‐CE content declined from 2.95 ± 0.24 μg/g DW at the mature green stage to 1.66 ± 0.31 μg/g DW at full ripeness (Table S9). Similarly, most transgenic lines displayed increased β‐CE content at the full ripe stage compared to mature green fruit. This was particularly evident across the Exp1‐*ZmPsy1* lines in the S crop where an average 49% increase in β‐CE was recorded. In contrast, fruit from Ubi‐*MtPsy2a* line FT294 displayed approximately 13.3%, 11.7% and 16.8% decline in β‐CE content between the mature green and full ripe stage in the S, SR1 and SR2 crops, respectively. A comparable, but less pronounced, decline was also observed in line FT324 which carries the same promoter‐transgene combination. High variation in the differences between β‐CE content in mature green compared to full ripe fruit across the vegetative seasons was observed for both Ubi‐*ZmPsy1* lines. In line FT287, β‐CE content at the full ripe stage was approximately 50% higher than in mature green fruit during the S and SR1 crops but decreased by 30% at full ripeness in the SR2. Conversely, line FT309 showed a 61% decrease in β‐CE at full ripeness in the S crop, no change in SR1, and a 91% increase in SR2.

### 2.3 Transgene Activity Does Not Alter the Expression of Endogenous Carotenoid Biosynthetic Genes in Banana Fruit

**FIGURE 3:**
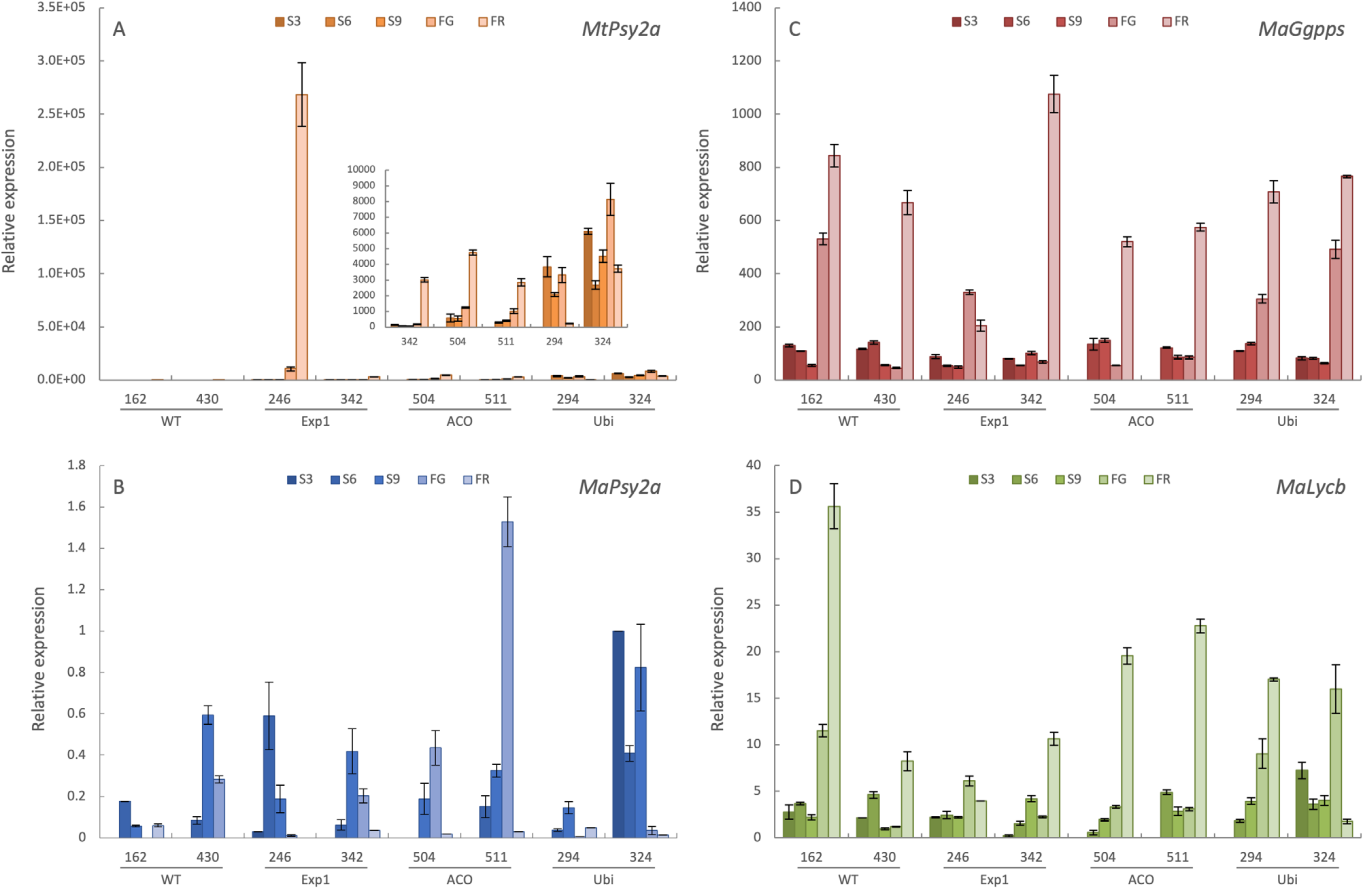
Carotenoid biosynthesis genes mRNA abundance during the development of the fruit of selected wild‐type and *MtPsy2a* transgenic banana lines. Relative transcript levels of *MtPsy2a* (A), *MaPsy2a* (B), *MaGgpps* (C) and *MaLycb* (D) were calculated using the ΔΔCT method, normalised with 2 reference genes and expressed relative to the *MaPsy2a* transcript levels in the S3 sample of line FT324. Lines FT162 and FT430 (wild‐type); FT246 and FT342 (Exp1‐*MtPsy2a*); FT504 and FT511 (ACO‐*MtPsy2a*) and FT294 and FT324 (Ubi‐*MtPsy2a*). S3, S6, S9 are 3, 6, 9 weeks post‐bunch emergence (PBE), respectively; FG, full green, FR, full ripe. Samples collected from the first ratoon crop of FT‐1.

Although elevated expression of the *PSY* transgene was necessary for enhanced carotenoid accumulation, it was not sufficient to explain the variation in pVA content observed across the different lines and cropping seasons (Figure 2). Therefore, analysis of the expression of key endogenous carotenoid biosynthetic genes was conducted in a subset of *MtPsy2a* expressing transgenic lines to determine whether transcriptional regulation of these genes contributed to the observed phenotypes. Expression of the *MtPsy2a* transgene in addition to the endogenous geranylgeranyl diphosphate synthase (*MaGgpps*), the final gene in the MEP pathway which produces the substrate for PSY, lycopene β‐cyclase (*MaLycb*), of which the encoded enzyme cyclises lycopene into β‐carotene and controls a key branch point in the carotenoid biosynthesis pathway, and the Cavendish homologue of *MtPsy2a*, *MaPsy2a*, was analysed across fruit development via qPCR. As expected, expression of the *MtPsy2a* transgene could not be detected in the wild‐type controls. For both the Exp1 and ACO promoter lines, expression of the *MtPsy2a* transgene increased across development and was highest at the full ripe stage (Figure 3A), consistent with GUS‐reporter data (Paul et al. 2017). Interestingly, transgene expression was found to be around 60‐ and 90‐fold greater at the full green and full ripe stages, respectively, in line FT246 compared to line FT342, despite both lines containing the Exp1‐*MtPsy2a* construct. However, this difference in expression only related to a ~12.2% increase in β‐CE at full green (FG) and 84.8% at full ripe (FR) in line FT 246 (Table 2) further supporting the idea that factors beyond transgene transcript levels influence pVA accumulation. Unlike Exp1‐regulated lines, ACO‐ and Ubi‐driven transgenes showed higher expression at earlier stages of fruit development. As ACO‐ and Ubi‐ driven lines accumulated higher β‐CE levels than Exp1 lines, this suggests that early metabolic activity may contribute to overall greater carotenoid accumulation. In contrast to ACO and Exp1 regulated lines, both Ubi‐*MtPsy2a* lines exhibited transgene expression which remained consistently high without showing a clear developmental trend, as expected for a constitutive promoter. The native Cavendish *MaPsy2a* showed no consistent expression pattern across fruit development and displayed high variation across all lines including both the wild‐type and in all transgenic backgrounds (Figure 3B), likely due to low overall expression of this gene in fruit. By contrast, *MaGgpps* and *MaLycb* showed relatively consistent expression patterns with the highest expression of both genes detected at the full ripe stage (Figure 3C,D). Expression of these genes did not appear effected by transgene addition in any of the lines investigated. As endogenous gene expression appears largely stable across genotypes, this indicates that additional regulatory mechanisms likely contribute to carotenoid accumulation in transgenic banana fruit.

**TABLE 2:** Accumulation of carotenoids during stages of fruit development.

Line ID	Promoter‐*transgene*	β‐carotene equivalents (μg/g DW)				
		S3	S6	S9	FG	FR
FT162	Wild‐type	1.41 ± 0.00	1.38 ± 0.00	1.11 ± 0.00	1.66 ± 0.00	2.05 ± 0.00
FT430		2.97 ± 0.00	1.58 ± 0.00	1.81 ± 0.00	1.65 ± 0.00	1.78 ± 0.00
FT246	Exp1‐*MtPsy2a*	4.10 ± 0.00	3.36 ± 0.00	3.76 ± 0.00	8.26 ± 0.00	8.48 ± 0.00
FT342		4.28 ± 0.00	2.36 ± 0.00	6.65 ± 1.00	7.42 ± 1.00	4.57 ± 0.00
FT504	ACO‐*MtPsy2a*	3.23 ± 0.00	6.74 ± 0.00	16.73 ± 1.00	12.14 ± 1.00	11.68 ± 1.00
FT511		7.59 ± 1.00	4.84 ± 1.00	5.08 ± 0.00	5.30 ± 0.00	5.95 ± 0.00
FT294	Ubi‐*MtPsy2a*	14.58 ± 2.00	7.31 ± 0.00	8.45 ± 1.00	13.54 ± 2.00	12.03 ± 1.00
FT324		17.88 ± 2.00	21.88 ± 2.00	27.99 ± 2.00	26.60 ± 2.00	33.44 ± 2.00

### 2.4 Environmental and Seasonal Effects on Carotenoid Accumulation in Wild‐Type and Transgenic Lines

Previous work by Paul et al. (2017) reported high variation in pVA content in the fruit from wild‐type lines and noted a seasonal trend, observing that fruit with shorter bunch filling durations, occurring during warmer months, accumulated lower levels of pVA. To explore this further, the effect of environmental and seasonal variables, specifically mean temperature, mean rainfall, and average global solar exposure, on pVA accumulation in the fruit of wild‐type and transgenic lines was investigated. The monthly data averaged for the growing season from 2012 to 2016 are presented in Table S10, along with graphs showing S, SR1 and SR2 harvests distribution between January 2013 and May 2015 (Figure S6). Banana fruits harvested during spring (September to November) and summer (December to February) were exposed to more global solar energy resulting in more daily light irradiation (20–23.6 MJ/m^2^) than fruits harvested in autumn (March to May) and winter (June to August) (14.1–18.1 MJ/m^2^) (Table S10). Additionally, fruits harvested in summer and autumn experienced heavier rainfall (174–624 mm) and concurrent higher temperatures (27°C–32°C) than fruits harvested in winter and spring (60–205 mm and 24°C–31°C respectively). These differences in environmental factors had a profound effect on fruit maturity (bunch filling time) and possibly fruit carotenoid biosynthesis and accumulation.

Confirming previous observations by Paul et al. (2017), a significant positive correlation between fruit β‐CE concentration and bunch filling time was found in fruit from wild‐type lines (*r* = 0.75, *p* = 0.02) (Table S11). Strong correlations between β‐CE and bunch filling time were also noted for several transgenic lines including Ubi‐*MtPsy2a* (*r* = 0.82, *p* = 0.002), ACO‐*ZmPsy1* (*r* = 0.74, *p* = 0.006) and ACO‐*ZmPsy1* co‐expressed with Exp1‐*PaCrtI* (*r* = 0.62, *p* = 0.04). In addition to bunch filling time, in wild‐type fruit a very strong negative correlation between β‐CE content and mean temperature was observed (*r* = −0.87, *p* = 0.002) whereas no significant correlations were found between β‐CE and mean rainfall or average global solar exposure. The inverse relationship between β‐CE content and mean temperature was also evident in all transgenic lines where a PSY homologue was regulated by the Exp1 promoter (Exp1‐*ZmPsy1*, *r* = −0.82, *p* = 0.001; Exp1‐*MtPsy2a*, *r* = −0.73, *p* = 0.02). Whilst a moderate significant correlation between β‐CE and mean temperature was seen in lines co‐expressing *ZmPsy1* with *PaCrtI* (*r* = −0.62, *p* = 0.04), this was not the case for any lines utilising the ACO or Ubi promoters. No significant correlations were found for β‐CE with bunch filling time or any environmental parameter for either the Ubi‐*ZmPsy1* or the ACO‐*MtPsy2a* lines suggesting that in the fruits of these transgenic lines, carotenoid accumulation may be decoupled from environmental and seasonal effects.

Given that bunch filling time was correlated with β‐CE accumulation in the wild‐type and several transgenic lines, the seasonal effects on bunch filling time were also investigated. A strong negative relationship between bunch filling and mean rainfall was identified in the wild‐type (*r* = −0.93, *p* < 0.005) and across all transgenic lines, with correlation coefficients ranging from −0.67 to −0.95 and all *p*‐values < 0.03 (Table S12) indicating that higher average rainfall was associated with shorter bunch filling periods in banana.

In addition to environmental variables, agronomic traits were also assessed for potential associations with β‐CE content through correlation analysis (Table S13). In wild‐type lines, fruit β‐CE content was significantly negatively correlated to plant height (*r* = −0.60, *p* = 0.02), bunch weight (*r* = −0.67, *p* = 0.006) and bunch finger number (*r* = −0.76, *p* = 0.001). Plant height was also negatively correlated to β‐CE in the transgenic lines Exp1‐*ZmPsy1* (*r* = −0.85, *p* < 0.0001), ACO‐*ZmPsy1* (*r* = −0.63, *p* = 0.03), Exp1‐*MtPsy2a* (*r* = −0.62, *p* = 0.03) and ACO‐*MtPsy2a* (*r* = −0.66, *p* = 0.008). Whilst no association between plant cycle time and fruit β‐CE content was evident in the wild‐type, a strong positive correlation was found in the Ubi‐*MtPsy2a* lines (*r* = 0.85, *p* = 0.0005) and moderate correlations observed for the ACO‐*ZmPsy1* (*r* = 0.64, *p* = 0.03) and Exp1‐*ZmPsy1* (*r* = 0.54, *p* = 0.04) lines. Consistent with the wild‐type, most transgenic lines exhibited a significant negative correlation between β‐CE and bunch weight, with the exception of Exp1‐*MtPsy2a*, which lacked a correlation between β‐CE content and finger number.

### 2.5 Characterising the ‘Golden’ Phenotypes Resulting From *ZmPsy1* Over‐Expression in Transgenic Bananas

**FIGURE 4:**
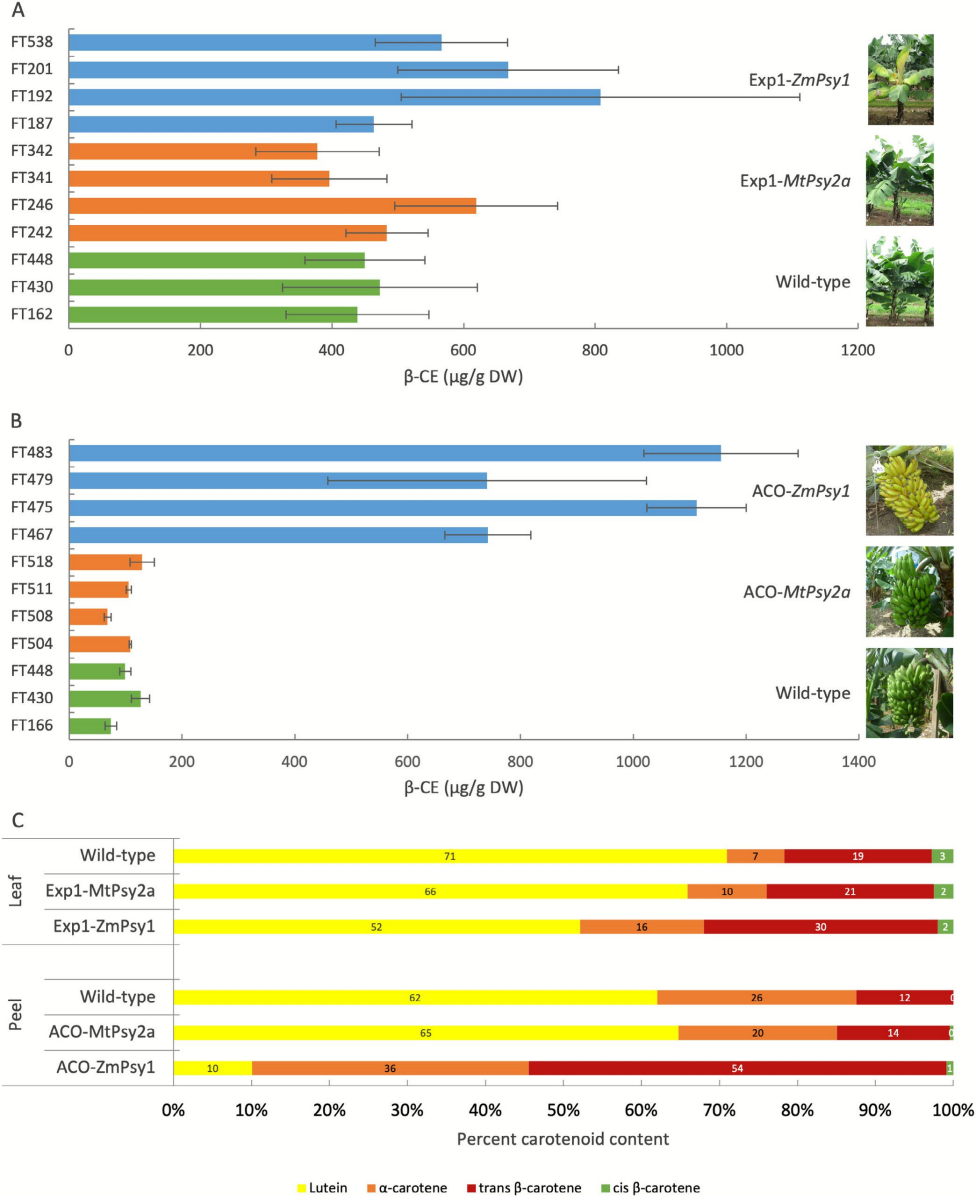
Carotenoid content and composition in the leaves and peel of wild‐type and transgenic ‘Cavendish’ banana lines with a ‘golden’ phenotype. Carotenoid content in banana leaf (A) and peel from mature unripe fruit (B) was measured by HPLC and the data presented is an average of 3 technical replicates from 4 biological replicates (*n* = 12) collected from the sucker crop (S) and expressed as β‐carotene equivalents (β‐CE) ± SD. The carotenoid composition is presented as an average from 4 biological replicates and expressed as percentage (C). Lines presented are: Wild‐type lines FT162, FT430 and FT448; Exp1‐*ZmPsy1* ‘golden’ leaf lines FT187, FT192, FT201 and FT538; Exp1‐*MtPsy2a* lines FT242, FT246, FT341 and FT342; ACO‐*ZmPsy1* ‘golden’ peel lines FT467, FT475, FT479 and FT483 and ACO‐*MtPsy2a* lines FT504, FT508, FT511 and FT518.

To elucidate the biochemical basis of the visually distinctive ‘golden’ phenotypes observed exclusively in the Exp1‐*ZmPsy1* lines, which produce golden leaves, and the ACO‐*ZmPsy1* lines, which produced unripe fruit with golden peels, β‐CE content and individual carotenoids were profiled in the corresponding tissues and compared to phenotypically normal lines. In the leaves of Exp1‐*ZmPsy1* lines, β‐CE content was elevated relative to controls (*p* = 0.04, two‐tailed *t*‐test) (Figure 4A; Table S14) which coincided with a 27% reduction in the proportion of lutein but a 158% and 228% increase in β‐ and α‐carotene, respectively (Figure 4C). This is in contrast to Exp1‐*MtPsy2a* lines, which did not exhibit the ‘golden’ leaf phenotype and showed no significant difference in β‐CE content compared to the controls. Amongst the Exp1‐*ZmPsy1* lines, the most pronounced ‘golden’ pigmentation was observed in line FT192. Interestingly, this line also displayed the highest proportion of α‐ and β‐carotene and the lowest proportion of lutein, present at only 22% compared to an average of 71% in the wild‐type (Table S14; Figure S5A).

In the peel of unripened ACO‐*ZmPsy1* fruit displaying the ‘golden’ phenotype, β‐CE content was, on average, 9.4‐fold higher than in wild‐type controls (Figure 4B; Table S15). In contrast, plants expressing the banana homologue *MtPsy2a* under the same ACO promoter developed green ‘true‐to‐type’ peels where β‐CE levels remained comparable to the wild‐type. The ACO‐*ZmPsy1* ‘golden’ phenotype was on average associated with a pronounced shift in carotenoid composition with the proportion of lutein decreasing from 62% in the wild‐type to just 10%, while the proportion of the pVA carotenoids α‐ and β‐carotene increased from 26% and 12% to 36% and 54%, respectively (Figure 4C). This was consistent in all four ACO‐*ZmPsy1* lines investigated (Figure S5B).

**FIGURE 5:**
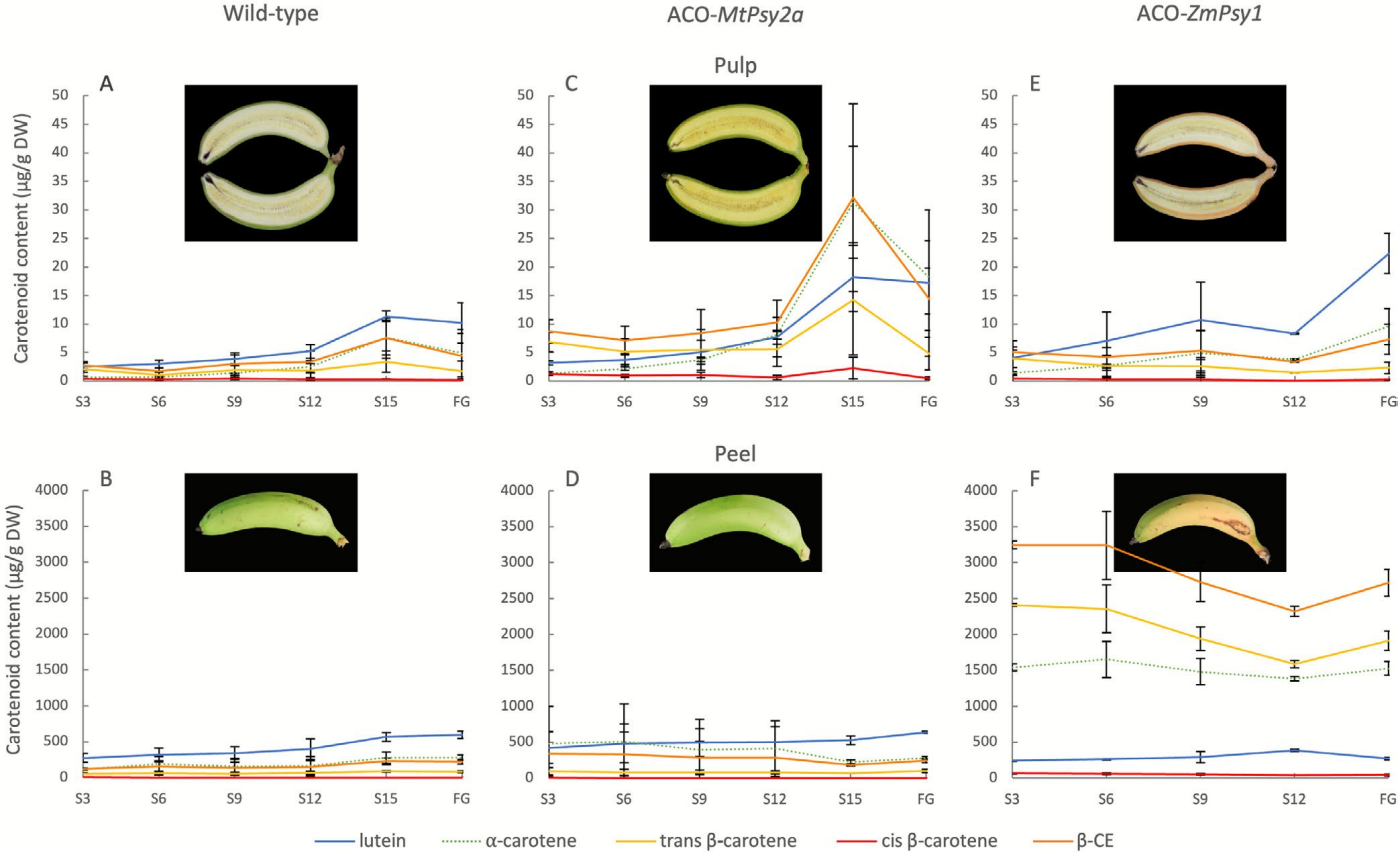
Carotenoid content in the pulp and peel during the development of wild‐type and transgenic ‘Cavendish’ banana fruit. Wild‐type lines (A and B); ACO‐*MtPsy2a* lines (C and D) and ACO‐*ZmPsy1* lines (E and F). Data represent means ± SD of 3 technical replicates from 3, 3 and 2 biological replicates from wild‐type, ACO‐*MtPsy2a and* ACO‐*ZmPsy1* lines respectively and collected from the sucker ratoon 2 crop (SR2). β‐CE, β‐carotene equivalents. S3, S6, S9, S12 and S15 represent 3, 6, 9, 12 and 15 weeks post‐bunch emergence, respectively. FG, full green.

To further investigate the onset and tissue specificity of the ‘golden’ peel phenotype, carotenoid profiles were assessed across fruit development in both ACO‐*ZmPsy1* and ACO‐*MtPsy2a* lines. In the pulp of wild‐type fruit, the carotenoid content gradually increased over development and remained at its highest from 15 weeks post‐bunch emergence (S15) to full maturity (Figure 5A; Table S16). A similar trend was observed for wild‐type peel, although this tissue accumulated over 50‐fold greater total carotenoid content compared to the pulp (Figure 5B; Table S17). In both wild‐type tissues, lutein was the most abundant carotenoid. In the pulp of ACO‐*MtPsy2a* fruit, elevated levels of each individual carotenoid were detected from the earliest developmental stage measured, 3 weeks post‐bunch emergence (S3), with peak accumulation at S15 (Figure 5C). Despite increased carotenoid accumulation in the pulp, ACO‐*MtPsy2a* peels remained phenotypically green. The average concentration of each carotenoid in this tissue was approximately 1.8‐fold higher than that of the wild‐type at all stages, except for S15, which showed an approximately 15% reduction (Figure 5D; Table S17). By the mature green stage there were no substantial differences observed between the two genotypes. In contrast, pulp of ACO‐*ZmPsy1* fruit showed lutein as the predominant carotenoid, with levels increasing progressively over development and reaching a maximum at full maturity, where total carotenoid content was approximately 2‐fold higher than the wild‐type (Figure 5E). ACO‐*ZmPsy1* peel displayed the striking ‘golden’ phenotype which coincided with markedly higher levels of α‐carotene (13‐fold increase), all‐*trans*‐β‐carotene (43‐fold increase) and *cis*‐β‐carotene (14‐fold increase) at the S3 developmental stage (Figure 5F). Unlike the pVA carotenoids, lutein levels in ACO‐*ZmPsy1* peel were comparable to the wild‐type and relatively stable over development. On the other hand, the pVA carotenoid concentrations declined from S3 until, followed by a modest increase towards full maturity.

## 3 Discussion

In this study, we conducted a comprehensive, multi‐generation field assessment of transgenic Cavendish banana lines engineered to enhance pVA accumulation in fruit. The primary objective of this study was not to advance the engineered lines as nutritional products but to characterise the stability and behaviour of the biofortification trait across successive vegetative generations under controlled, well‐replicated field conditions. Monitoring agronomic performance, visible phenotypes and pVA accumulation across cycles allowed us to assess how the engineered trait responds to environmental, developmental and promoter‐isoform variables. Establishing trait stability in the well‐characterised Cavendish background is a necessary step towards deployment in the intended target EAHB cultivars, where consistent expression across generations will be essential for nutritional impact. Studies investigating trait stability in vegetatively propagated crops are relatively scarce, however examples include reports on potatoes (Pence et al. 2024), strawberries (Cervantes et al. 2020), cassava (Oyelakin et al. 2015), sugarcane (Harrison et al. 2014; Kinkema et al. 2014; Caffall et al. 2017; Yao et al. 2017), synthetic apomictic rice (Liu et al. 2023) and banana (Tripathi et al. 2014). The scarcity of multi‐season, multi‐generation datasets in banana represents not only a scientific gap but also a regulatory one. Regulatory authorities require evidence that an introduced trait is stable, predictable and does not cause unintended agronomic effects across vegetative generations. For vegetatively propagated crops such as banana, where clonal continuity underpins deployment, such evidence is essential. The data presented here provide a foundational guide for what future regulatory assessments may expect from multi‐season field trials, addressing key requirements for demonstrating trait stability, environmental responsiveness and agronomic comparability in pVA‐enhanced banana.

Amongst the key findings of this study was a consistent increase in β‐CE content in the S crop compared with the initial plant crop across all lines, including the wild‐type. The plant crop (from which all suckers were collected), representing the first harvest after field establishment of tissue‐culture‐derived plants, reflects a phase in which plants are still adapting to field conditions and may be constrained by establishment‐related stresses that limit their metabolic performance. By contrast, sucker crops (S, SR1, and SR2) developed from already established mother plants and therefore grew under more favourable physiological conditions, enabling greater accumulation of pVA carotenoids. This pattern is consistent with observations in other clonally propagated crops, where trait performance often improves in the first clonal generations. For instance, in the banana cultivar ‘Sukali Ndiizi’ (AAB group) genetically modified for resistance to banana *Xanthomonas* wilt disease, resistance increased from 85%–93% in the mother plants to 100% in the first ratoon plants, an effect attributed to the physiological stress of mother plants adapting from tissue culture to field conditions (Tripathi et al. 2014). Comparable improvements in early ratoon crops have also been observed in genetically modified sugarcane, with increased reporter gene expression (Kinkema et al. 2014) and enhanced accumulation of recombinant enzymes such as fungal cellobiohydrolase I and II and bacterial endoglucanase (Harrison et al. 2014). Conversely, other studies in sugarcane have reported stable recombinant protein production across successive vegetative generations (Caffall et al. 2017), highlighting that trait performance across clonal generations may vary depending on the transgene and expression context.

Another important finding of this study was the progressive reduction in fruit pVA accumulation in SR1 and SR2 crops relative to the original S crop. Unlike the plant‐to‐sucker crop comparison, these differences cannot be attributed to tissue‐culture‐related stress, as all harvests were obtained from well‐established field plants. Instead, the data strongly indicate a seasonal influence on pVA content. In previous work, high variation in wild‐type fruit pVA levels was shown to follow a seasonal trend, with shorter bunch filling durations during warmer months associated with reduced pVA accumulation (Paul et al. 2017). The present findings are consistent with this observation: most S crop fruit were harvested during winter (July–September), whereas SR1 and SR2 harvests occurred between March–May and December–April, respectively, periods characterised by warmer conditions that coincided with reduced pVA levels. The most plausible explanation is that the effect is temperature driven. Elevated temperatures accelerate fruit filling, whereas cooler, suboptimal conditions slow the process. This interpretation is also supported by the strong negative correlation between β‐CE accumulation and mean temperature observed in wild‐type fruit in this study. Consequently, fruit harvested at the same calliper grade are not developmentally equivalent: those developing under cooler conditions are physiologically more advanced than those from warmer months. Reduced pVA accumulation in fruit harvested during warmer periods is therefore most likely a consequence of earlier developmental status at the time of harvest.

The influence of temperature on pVA content may not be limited to developmental timing but could also involve direct effects on carotenoid metabolism. Elevated temperatures may impair the biosynthesis of carotenoids or reduce their stability during fruit filling, providing an additional explanation for the reduced accumulation observed in wild‐type fruit under warmer conditions. Interestingly, in transgenic lines expressing *ZmPsy1* or *MtPsy2a* under the control of either the ACO or Ubi promoter, no correlation between β‐CE accumulation and mean temperature was detected. This suggests that the introduced transgenes may buffer carotenoid accumulation against environmental variation, effectively bypassing regulatory constraints that govern the native pathway.

To assess whether the introduction of *PSY* transgenes affected the native carotenoid biosynthetic pathway, the expression of several key endogenous genes was examined. However, no differences in *MaLycb*, *MaPsy2a*, or *MaGgpps* expression was found in transgenic lines compared to controls. These findings suggest that factors beyond transgene expression levels, such as post‐transcriptional regulation, protein–protein interactions, enzyme activity or carotenoid sequestration, may contribute to the observed variation in carotenoid accumulation. Recognising this complexity, current pVA biofortification strategies increasingly employ multifaceted approaches to enhance carotenoid accumulation by combining upregulation of early biosynthetic gene/(s) with genetic strategies aimed at enhancing carotenoid sequestration, reducing carotenoid turnover or stabilising biosynthetic enzymes (Watkins and Pogson 2020). In a previous study, authors used CRISPR/Cas9 gene editing to disrupt *carotenoid cleavage dioxygenase 4* (*CCD4*), a gene known to initiate carotenoid catabolism, in the Rasthali banana cultivar (AAB genome). The resultant edited plants showed a modest increase in β‐carotene levels in the roots (2.3–2.7‐fold increase) and leaves (1.3–1.4‐fold increase) compared to controls (Awasthi et al. 2022). Whilst the authors did not report data on carotenoid concentration in the fruit of edited lines, combining *CCD4* gene editing with *PSY* over‐expression could represent a promising strategy for further enhancing pVA content in bananas.

A consistent pattern observed in this study was a negative correlation between β‐CE content and bunch weight, suggesting that pVA accumulation may impose inherent metabolic trade‐offs. Similar observations have been reported across multiple carotenoid‐biofortified crops. In transgenic wheat, elevated carotenoid levels were associated with reduced seed weight (Cong et al. 2009), while transgenic cassava lines showed decreased storage‐root dry matter content (Beyene et al. 2018) and biofortified maize displayed yield reductions under field conditions (Zhu et al. 2008). Yield penalties have also been noted in conventionally bred orange‐fleshed sweet potato, where β‐carotene content is negatively correlated with starch and dry‐matter content (Cervantes‐Flores et al. 2011; Mahaman Mourtala et al. 2023), likely reflecting shared metabolic constraints or historical selection biases within germplasm pools. More recently, Buah et al. (2025) demonstrated that EAHB over‐expressing *MtPsy2a* also exhibited reduced yield. Importantly, such trade‐offs are not restricted to pVA biofortification. Iron and zinc biofortification efforts in rice (Moreno‐Moyano et al. 2016) and banana (Cooper et al. 2025) similarly reported penalties in yield or key yield components, underscoring the broader challenge of achieving nutritional enhancement without compromising agronomic performance.

Several mechanistically plausible pathways explain why elevated pVA accumulation can impose yield penalties in starchy crops. One widely supported mechanism is direct competition for shared metabolic precursors (Villwock et al. 2024). Carotenoid biosynthesis draws heavily on glyceraldehyde‐3‐phosphate (G3P), pyruvate and ATP to supply the methylerythritol phosphate (MEP) pathway, diverting carbon and energy away from starch biosynthesis (Moise et al. 2014). A such, high‐carotenoid genotypes of sweet potato, cassava and maize engineered to over‐express *DXS*, *crtB*, *PSY* or *crtI* consistently show increased glycolytic flux, downregulation of ADP‐glucose pyrophosphorylase and starch synthase, elevated sucrose and fatty acids, and reduced starch accumulation (Decourcelle et al. 2015; Beyene et al. 2018; Drapal et al. 2022). In cassava, this metabolic reallocation also decreased dry matter content by reducing starch synthesis and increasing starch degradation (Beyene et al. 2018). Similar metabolic signatures have been reported in banana, where high‐carotenoid cultivars exhibit upregulation of glycolysis‐related proteins and metabolites, together with increased soluble sugar content compared to low‐carotenoid varieties (Heng et al. 2019). These findings demonstrate that increasing carotenoid flux can reduce carbon available for storage carbohydrates, a likely contributor to the bunch weight reductions observed in some pVA‐enhanced bananas line in our study. Other potential mechanistic pathways to reduced yield include plastid remodelling, apocarotenoid‐ and sugar‐mediated signalling effects, and the increased metabolic cost of carotenoid sequestration structures, as described in a review by Villwock et al. (2024). These interacting processes provide coherent physiological rationales for the line‐dependent yield penalties observed in some pVA‐enhanced bananas, and explain the negative correlation between β‐CE content and bunch weight observed in this and other biofortification studies.

Beyond yield, several transgenic lines in this study displayed multiple agronomic traits that differed significantly from the wild‐type, indicating that enhanced pVA accumulation can have broader physiological consequences. In the Exp1‐*ZmPsy1* lines for instance, strong negative correlations between β‐CE content and both plant height (*r* = −0.85, *p* < 0.0001) and finger length (*r* = −0.75, *p* = 0.001) suggest coordinated reductions in vegetative and reproductive traits. Such patterns are consistent with pleiotropic effects of *PSY* over‐expression, potentially arising from perturbation of hormonal signalling pathways. The first committed step of gibberellic acid (GA) biosynthesis shares the precursor geranylgeranyl diphosphate (GGPP) with carotenoid biosynthesis, and increased flux towards carotenoid formation may reduce GGPP availability for GA production. Supporting this mechanism, transgenic tomato plants constitutively over‐expressing PSY display dwarf phenotypes and a roughly 30‐fold reduction in active GA₁ levels (Fray et al. 1995). Redirecting carbon and isoprenoid intermediates towards carotenoid biosynthesis also incurs metabolic costs that may underlie the line‐dependent reductions in bunch weight and vigour observed here. GGPP is the central isoprenoid precursor not only for carotenoids and GAs but also for chlorophylls, and competition for this substrate can disrupt isoprenoid balance (Nisar et al. 2015). In tomato fruit engineered for elevated phytoene, enhanced flux through PSY perturbed plastid development, altered intermediary metabolism and induced the formation of chromoplast‐like structures (Fraser et al. 2007). More broadly, *PSY* over‐expression has been associated with reduced growth or dwarf phenotypes in tomato due to competition between carotenoid and GA biosynthesis (Giuliano et al. 2008).

Taken collectively, these mechanistic insights offer potential explanations for the reductions in vigour, bunch weight and specific morphological traits observed in a subset of Ubi and Exp1 constructs, emphasising that pVA enhancement can influence multiple physiological processes beyond yield alone. Additional measurements of starch, sugars and dry matter in the EAHB lines now under evaluation will help determine whether altered carbon allocation also contributes to the growth effects reported here and by Buah et al. (2025).

Beyond the current study and earlier work by Paul et al. (2017), ‘golden’ leaf phenotypes have only been reported after virus‐mediated expression of the bacterial *PSY* gene, *CrtB*, in lettuce and *Nicotiana benthamiana* leaves (Llorente et al. 2020; Morelli et al. 2024). In these studies, an increase in carotenoid content, particularly β‐carotene, was accompanied by a chloroplast‐to‐chromoplast transition which included dismantling of photosynthetic structures, loss of photosynthetic activity, and proliferation of carotenoid sequestration structures called plastoglobules. Interestingly, over‐expression of the Arabidopsis, maize or rice homologues of *PSY* in Arabidopsis leaves did not have the same effect (Álvarez et al. 2016), suggesting that the chloroplast‐to‐chromoplast transition may be specific to certain PSY isoforms and not solely dependent on elevated *PSY* transcripts. The importance of the transgene origin in achieving enhanced pVA with minimal metabolic disruptions was highlighted during the development of Golden Rice 2, where the source of *PSY* was critical to the project success (Paine et al. 2005). As ‘golden’ phenotypes were only observed in *ZmPsy1*‐expressing lines and not those expressing *MtPsy2a* in this study, a cisgenic strategy, using *MtPsy2a* alongside banana‐derived regulatory elements, was considered a more effective approach to achieve nutritionally significant pVA carotenoids accumulation whilst preserving agronomic characteristics, thereby increasing the likelihood of successful deployment of pVA enhanced EAHBs.

For nutritional context, the Banana21 programme established a working target of 20 μg/g DW β‐carotene equivalents (β‐CE) in EAHB fruit, a level predicted to raise contributions to the Estimated Average Requirement (EAR) for vitamin A from roughly 25% to around 50% for young children (Paul et al. 2017). Several promoter‐isoform combinations evaluated in this study, particularly Ubi‐*MtPsy2a*, Ubi‐*ZmPsy1* and ACO‐*MtPsy2a*, routinely exceeded these thresholds in Cavendish. Although Cavendish is not the intended deployment background, these data demonstrate that the engineering strategy can achieve nutritionally meaningful β‐CE levels when translated into preferred EAHB cultivars.

Importantly, the recent study by Buah et al. (2025), successfully demonstrated that over‐expressing *MtPsy2a* into two locally preferred EAHB cultivars, M9 and Nakitembe, can enhance pVA accumulation in fruit from these plants. Transgenic lines accumulated greater than 3‐fold higher β‐CE content compared to controls and showed no differences in crop cycle time. As several lines have been selected for national agronomic performance trials, these findings represent a major step towards the release of nutritionally enhanced EAHB varieties in Uganda.

Together, these results show that pVA enhancement in banana can be stably expressed across generations and environments, and that promoter‐transgene combinations differ markedly in both their nutritional performance and agronomic effects. While some lines exhibited reduced vigour or bunch weight, the mechanistic considerations outlined above offer plausible explanations and highlight opportunities for further optimisation. The identification of construct architectures capable of achieving nutritionally meaningful pVA levels, coupled with predictable multi‐season behaviour, provides a strong foundation for advancing the most effective combinations into EAHB backgrounds. Overall, this work advances understanding of carotenoid biofortification strategies in banana and identifies construct designs with clear potential for continued development in target EAHB cultivars.

## 4 Experimental Procedures

### 4.1 Vectors Construction

The suite of seven pBMGF‐DC pVA‐enhancement binary transformation constructs reported in this study are exactly as described in Paul et al. (2017) and detailed in Figure S1. Briefly, the banana (*MtPsy2a*; GenBank #: JX195659) and maize (*ZmPsy1*; GenBank #: U32636) phytoene synthase genes were put under the transcriptional control of either the banana expansin (pBMGF‐DC‐97 and ‐100), the banana 1‐aminocyclopropane‐1‐carboxylate oxidase (pBMGF‐DC‐98 and ‐101) or the maize polyubiquitin1 (pBMGF‐DC‐99 and ‐102) promoters, respectively. The *Pantoea ananatis* phytoene desaturase (*PaCrtI*; Genbank #: D90087) gene was only expressed by the banana expansin promoter (pBMGF‐DC‐104). Every expression cassette used the 3′ untranslated region (3′ UTR) of the nopaline synthase gene (*Nos*) from *Agrobacterium tumefaciens* (Depicker et al. 1982).

### 4.2 Plant Transformation and Regeneration

Transgenic plants of the cultivar Dwarf Cavendish (AAA Group, Cavendish Subgroup) with enhanced fruit pVA had previously been generated from embryogenic cell suspensions (ECS) using *Agrobacterium*‐mediated transformation (Paul et al. 2017).

### 4.3 Banana Field Trial Design and Conditions

All transgenic and wild‐type ‘Cavendish’ (AAA) banana lines used in this study were previously growing in field trial 1 (FT‐1) at the Queensland Department of Primary Industries (DPI), South Johnstone Research Facility, Australia (Paul et al. 2017). Field trial 2 (FT‐2) included 27 independent transgenic and five wild‐type lines selected from FT‐1 and re‐planted in replicates of 10 plants (field derived suckers) in a randomised plot design (2 plots x 5 plants). Field trial conditions and maintenance were as previously described (Paul et al. 2017). FT‐2 was monitored for three consecutive crop cycles between 2012 and 2017.

### 4.4 Collection and Processing of Plant Samples for Analysis

Fruits were harvested at mature green (full green, FG) and where needed at 3, 6, 9, 12 and 15 weeks post‐bunch emergence (S3, S6, S9, S12 and S15) from each plant and delivered within 48 h of harvest. Fruits were processed either immediately or at 7 days post‐exposure (24 h) to ethylene for full ripe analysis (Paul et al. 2017). Banana leaf samples from the field were collected from the first fully expanded leaf prior to bunch emergence. Prior to further analysis, all samples were freeze‐dried in a Benchtop 4 K Freeze Dryer (VirTis) and homogenised in a Mini‐Beadbeater‐8 (Biospec Products). Fruit and leaf samples were processed under dim light conditions.

### 4.5 Quantification of Carotenoids Content

Total carotenoids were extracted from homogenised freeze‐dried banana pulp (200 mg), peel (25 mg) and leaf (25 mg) material and subsequently analysed by HPLC as described by Paul et al. (2017). Amounts of individual carotenoids (lutein, α‐carotene, *trans* and *cic* β‐carotene) as well as total carotenoids, pVA carotenoids and β‐CE are reported in μg/g DW.

### 4.6 Nucleic Acid Isolation

Genomic DNA (gDNA) for PCR and Southern blot analysis was isolated from 50 mg of homogenised freeze‐dried banana leaf tissue using a modified CTAB method (Stewart Jr. and Via 1993). Total RNA was isolated from 50 mg of homogenised freeze‐dried banana fruit pulp and peel using a protocol adapted from Valderrama‐Cháirez et al. (2002) where the tissue to extraction buffer ratio was increased to 8, and an additional centrifugation step (18 000 x *g* for 5 min) was added prior to the initial solvent extraction and phenol was omitted from all extraction steps. Isolation of plasmid DNA (pDNA) was done using the Wizard Plus SV Minipreps DNA Purification System (Promega).

### 4.7 DNase Treatment and Synthesis of Complementary DNA (cDNA)

Prior to cDNA synthesis, 3 μg of total RNA was DNase treated using the RQ1 RNase‐free DNase Kit (Promega). DNA‐free RNA samples (1.8 μg) were reverse‐transcribed into cDNA in 25 μL reactions using oligo(dT)_18_ primers and the GoScript Reverse Transcription System (Promega) according to the manufacturer's instructions. All cDNA samples for reverse transcriptase PCR (RT‐PCR) and quantitative RT‐PCR (qRT‐PCR) were diluted 1:10 (v/v) or 1:8 (v/v) in RNase‐free water, respectively. To ensure complete removal of gDNA contamination from our samples prior to RT‐ and qRT‐PCR, the presence of the cyclophilin (*CYP*) housekeeping gene was detected in all total RNA, DNase‐treated RNA, and cDNA samples as standard.

### 4.8 Polymerase Chain Reaction (PCR)

All routine end‐point PCR reactions were performed using the GoTaq Green master mix (Promega) following standard conditions provided by the manufacturer. Reaction mixes for PCR and RT‐PCR contained 1 x GoTaq Green master mix, 0.25 μM of individual primers (Table S1), 2 μL of template nucleic acid (gDNA, pDNA or diluted cDNA), and nuclease‐free water in a final volume of 20 μL. Thermal cycling conditions consisted of an initial denaturation step at 94°C for 2 min followed by 35 cycles of 94°C for 20 s, 55°C–62°C (primer set dependent) for 30 s, and 72°C for 1 min per kbp of expected PCR amplicon size, followed by a final extension for 5 min at 72°C.

### 4.9 Quantitative Real‐Time PCR (qRT‐PCR)

Gene expression was quantified by qRT‐PCR performed on the CFX384 Real‐Time Detection System (Bio‐Rad) using the GoTaq qPCR Master Mix (Promega) at 95°C for 2 min, followed by 45 cycles of 95°C for 10 s, 57°C for 30 s, 65°C for 5 s and 95°C for 5 s. Fluorescence was recorded in real time and detected at 470 nm while PCR product melting curves were analysed at 65°C–95°C after 45 cycles to confirm the specificity of the amplicon from each primer set. Each sample was analysed in three technical replicates in addition to inclusion of ‘no template’ and ‘RT negative’ controls. Relative expression levels were calculated using the CFX Manager 3.1 (Bio‐Rad) software and the 2^−ΔΔCT^ method (Livak and Schmittgen 2001; Schmittgen and Livak 2008). Ct values of target gene of interest were normalised using Ct values from the two stable reference genes cyclophilin (*CYP*) and ribosomal protein S2 (*RPS2*). All primers were designed with the Custom Primers‐OligoPerfect Designer software (Thermo Fisher Scientific) (Table 1).

### 4.10 Environmental Data Collection

Fruit pVA concentrations and respective bunch filling time for all samples harvested during the period of 2012 and 2016 were first grouped by respective constructs and then allocated to their respective month of harvest. An average of both parameters was then calculated for each month and compared to three environmental parameters (temperature (°C), rainfall (mm), and solar exposure (MJ/m^2^)) derived from the Australia Government, Bureau of Meteorology, all averaged monthly for the same period (http://www.bom.gov.au/climate/data).

### 4.11 Statistical Analysis

Statistical comparisons were made using the IBM SPSS Statistics Version 22 software package. Following Welch and Brown‐Forsythe robust tests of equality of means, mean differences were compared using one‐way analysis of variance (ANOVA) with Tukey's HSD (honest significant difference) post hoc test with statistical difference reported at 95% confidence level (*p* < 0.05). To assess relationships between environmental variables, β‐CE and agronomic properties, Pearson and Spearman correlations were calculated using Prism 10.4.1 (GraphPad, CA, USA).

## Author Contributions

J.L.D. and R.H. conceived and directed the project. R.H. and J.‐Y.P. designed the field trial. J.D., J.M.T. and J.‐Y.P. were involved in field trial collection and analysis. J.M.T. generated all the data under the supervision of J.‐Y.P., C.M. and B.M. J.‐Y.P., J.L.W., and J.M.T. analysed the data and wrote the manuscript. All authors reviewed the manuscript.

## Funding

This work was financially supported by the Bill & Melinda Gates Foundation, the Department for International Development (United Kingdom) and the Department of Foreign Affairs and Trade (DFAT—Australia) through the Australia Awards Scholarships.

## Conflicts of Interest

The authors declare no conflicts of interest.

## Supporting information


**Figure S1:** Schematic representation of binary vectors used for the genetic transformation of banana ECS. Nos, nopaline synthase; CaMV 35S, Cauliflower mosaic virus 35S; ACO, ACC oxidase promoter; Exp1, expansin 1 promoter; Ubi, maize polyubiquitin 1 promoter; *nptII*, neomycin phosphotransferase selectable marker gene; *MtPsy2a*, phytoene synthase 2a gene from the Fe′i banana ‘Asupina’; *ZmPsy1*, phytoene synthase 1 gene from maize ( *Zea mays* ) and *PaCrtI*, *Pantoea ananatis* phytoene desaturase.
**Table S1:** Selected pVA‐biofortified ‘Cavendish’ banana lines investigated in this study.
**Table S2:** Oligonucleotide primer names and sequences.
**Figure S2:** PCR detection of transgene(s) in selected transgenic ‘Cavendish’ bananas lines. The quality of the extracted genomic DNA was assessed by PCR amplification of the *CYP* housekeeping gene (A and B); detection of residual Agrobacterium contamination by PCR amplification of the *virC* gene from the AGL1 strain of *Agrobacterium tumefaciens* (C and D). The presence of the *ZmPsy1* (E and F), *PaCrtI* (G) and *MtPsy2a* (H) transgenes was detected using transgene specific primer sets. M; HyperLadder 1 kb marker; AGL1, wild‐type *A. tumefaciens* strain AGL‐1; AGL1‐P1, pOpt‐EBX recombinant AGL‐1; cDNA, complementary DNA; H2O, water control; P99, pBMGF‐DC‐99; P102, pBMGF‐DC‐102 and P104, pBMGF‐DC‐104.
**Figure S3:** Detection of transgene mRNA by RT‐PCR. DNA contamination was detected by PCR amplification of the *CYP* housekeeping gene in the total RNA extracts (A) and in the DNase treated extracts (B). The quality of cDNA synthesis was assessed by PCR amplification of a *CYP* mRNA transcript (C) followed by detection of transgene specific mRNA (D), *MtPsy2a* (top panel), *ZmPsy1* (middle panel), *PaCrtI* (bottom panel left) and *nptII* (bottom panel right). M; HyperLadder 1 kb marker; gDNA, genomic DNA; H2O, water control; P99, pBMGF‐DC‐99; P100, pBMGF‐DC‐100 and P104, pBMGF‐DC‐104.
**Table S3:** Number of T‐DNA‐integrated copies determined by Southern blot analysis.
**Table S4:** Agronomical characteristics of the sucker crop.
**Table S5:** Agronomical characteristics of the sucker ratoon 1 crop.
**Table S6:** Agronomical characteristics of the sucker ratoon 2 crop.
**Table S7:** Number of fruit samples collected and analysed by HPLC across three generations.
**Table S8:** Carotenoid content in the mature green fruit of transgenic Cavendish banana lines across four generations.
**Figure S4:** Percentage carotenoid composition in the green fruit of wild‐type and transgenic ‘Cavendish’ banana lines across 3 successive generations. Lines FT167 and FT430 are wild‐type while transgenic lines FT246, FT341 and FT342 (Exp1‐*MtPsy2a*); FT497 and FT518 (ACO‐*MtPsy2a*); FT295 and FT324 (Ubi‐*MtPsy2a*); FT187 and FT192 (Exp1‐*ZmPsy1*); FT467, FT475 and FT479 (ACO‐*ZmPsy1*); FT584, FT585 and FT587 (ACO‐*ZmPsy1* + Exp1‐*PaCrtI*); FT287 and FT309 (Ubi‐*ZmPsy1*). S, sucker crop; SR1, sucker ratoon 1 crop and SR2, sucker ratoon 2 crop. Data presented is an average of 3 technical replicates for 4 biological replicates (*n* = 12) expressed as percentages.
**Table S9:** Carotenoid content in the full ripe fruit of transgenic Cavendish banana lines across four generations.
**Figure S5:** Carotenoid composition in the leaves and peel of wild‐type and transgenic ‘Cavendish’ banana lines with a ‘golden’ phenotype. Carotenoid content in banana leaf (A) and peel from mature unripe fruit (B) was measured by HPLC in samples collected from the sucker crop and the data presented is an average from 4 biological replicates (with 3 technical replicates *n* = 12) expressed as percentage. Lines presented are: wild‐type lines FT162, FT430 and FT448; Exp1‐*ZmPsy1* ‘golden’ leaf lines FT187, FT192, FT201 and FT538; Exp1‐*MtPsy2a* lines FT242, FT246, FT341 and FT34; ACO‐*ZmPsy1* ‘golden’ peel lines FT467, FT475, FT479 and FT483 and ACO‐*MtPsy2a* lines FT504, FT508, FT511 and FT518.
**Table S10:** Environmental conditions influencing green mature fruit carotenoid accumulation and bunch filling time in wild‐type and transgenic ‘Cavendish’ banana.
**Figure S6:** Number of harvests for the sucker (S), sucker ratoon 1 (SR1) and sucker ratoon 2 (SR2) crops analysed in this study.
**Table S11:** Influence of environmental conditions on the accumulation of carotenoids in the green mature fruit of wild‐type and transgenic ‘Cavendish’ banana calculated via two‐tailed Pearson correlation.
**Table S12:** Influence of environmental conditions on bunch filling time in wild‐type and transgenic ‘Cavendish’ banana calculated via two‐tailed Spearman correlation.
**Table S13:** Correlations—Influence of green mature fruit carotenoid content on agronomical characteristics in wild‐type and transgenic ‘Cavendish’ banana calculated via two‐tailed Pearson correlation.
**Table S14:** Carotenoid content in the leaves of selected wild‐type and transgenic ‘Cavendish’ banana.
**Table S15:** Carotenoid content in the peel of selected wild‐type and transgenic ‘Cavendish’ banana.
**Table S16:** Carotenoid content in the pulp during the development of wild‐type and transgenic ‘Cavendish’ banana fruits.
**Table S17:** Carotenoid content in the peel during the development of wild‐type and transgenic ‘Cavendish’ banana fruits.

## Data Availability

The data that support the findings of this study are available on request from the corresponding author. The data are not publicly available due to privacy or ethical restrictions.
