# Supplementary material for: Pro‐Vitamin A Biofortified Cavendish Banana: Trait Stability in the Field
Source: Plant Biotechnol J. 2026 Feb 3;24(5):3327–44. doi: 10.1111/pbi.70516 (PMC13110172; doi:10.1111/pbi.70516)
Supplement: Supplementary file 1 — Figure S1: Schematic representation of binary vectors used for the genetic transformation of banana ECS. Nos, nopaline synthase; CaMV 35S, Cauliflower mosaic virus 35S; ACO, ACC oxidase promoter; Exp1, expansin 1 promoter; Ubi, maize polyubiquitin 1 promoter; nptII, neomycin phosphotransferase selectable marker gene; MtPsy2a, phytoene synthase 2a gene from the Fe′i banana ‘Asupina’; ZmPsy1, phytoene synthase 1 gene from maize ( Zea mays ) and PaCrtI, Pantoea ananatis phytoene desaturase. Table S1: Selected pVA‐biofortified ‘Cavendish’ banana lines investigated in this study. Table S2: Oligonucleotide primer names and sequences. Figure S2: PCR detection of transgene(s) in selected transgenic ‘Cavendish’ bananas lines. The quality of the extracted genomic DNA was assessed by PCR amplification of the CYP housekeeping gene (A and B); detection of residual Agrobacterium contamination by PCR amplification of the virC gene from the AGL1 strain of Agrobacterium tumefaciens (C and D). The presence of the ZmPsy1 (E and F), PaCrtI (G) and MtPsy2a (H) transgenes was detected using transgene specific primer sets. M; HyperLadder 1 kb marker; AGL1, wild‐type A. tumefaciens strain AGL‐1; AGL1‐P1, pOpt‐EBX recombinant AGL‐1; cDNA, complementary DNA; H2O, water control; P99, pBMGF‐DC‐99; P102, pBMGF‐DC‐102 and P104, pBMGF‐DC‐104. Figure S3: Detection of transgene mRNA by RT‐PCR. DNA contamination was detected by PCR amplification of the CYP housekeeping gene in the total RNA extracts (A) and in the DNase treated extracts (B). The quality of cDNA synthesis was assessed by PCR amplification of a CYP mRNA transcript (C) followed by detection of transgene specific mRNA (D), MtPsy2a (top panel), ZmPsy1 (middle panel), PaCrtI (bottom panel left) and nptII (bottom panel right). M; HyperLadder 1 kb marker; gDNA, genomic DNA; H2O, water control; P99, pBMGF‐DC‐99; P100, pBMGF‐DC‐100 and P104, pBMGF‐DC‐104. Table S3: Number of T‐DNA‐integrated copies determined by Southern blot analysis. Table [file PBI-24-3327-s001.docx]

**Supplementary Material**

**Pro-vitamin A biofortified Cavendish banana: trait stability in the field**

Jimmy M. Tindamanyire^1,2,3^, Jacinta L. Watkins^1^, Cara Mortimer^1,4^, Bulukani Mlalazi^1^, Jeff Daniells^5^, Rob Harding^1^, James L. Dale^1^, Jean-Yves Paul^1^🖂

^1^ Centre for Agriculture and the Bioeconomy, Queensland University of Technology, Brisbane, 4001, Queensland, Australia

^2^National Agricultural Research Laboratories, National Agricultural Research Organisation, Kampala, Uganda

^3^Current address: Department of Biological Sciences, Faculty of Science, Kabale University, P.O Box 317, Kabale, Uganda

^4^ Current address: IP Australia, 47 Bowes Street, Phillip, 2606, Australian Capital Territory, Australia

^5^Agri-Science Queensland, Department of Primary Industries, South Johnstone, Queensland, Australia

**Author details**:

tindajm@gmail.com; ORCID: 0000-0002-3686-7998

jacinta.watkins@qut.edu.au; ORCID: 0000-0002-7200-1331

cara.mortimer@hotmail.co.uk; ORCID: 0000-0003-2170-9556

bulukani.mlalazi@gmail.com; ORCID: 0000-0003-2959-2911

jeff.daniells@dpi.qld.gov.au

r.harding@qut.edu.au; ORCID: 0000-0002-9600-7287

j.dale@qut.edu.au; ORCID: 0000-0002-7910-5707

jy.paul@qut.edu.au; ORCID: 0000-0003-3246-2210 🖂


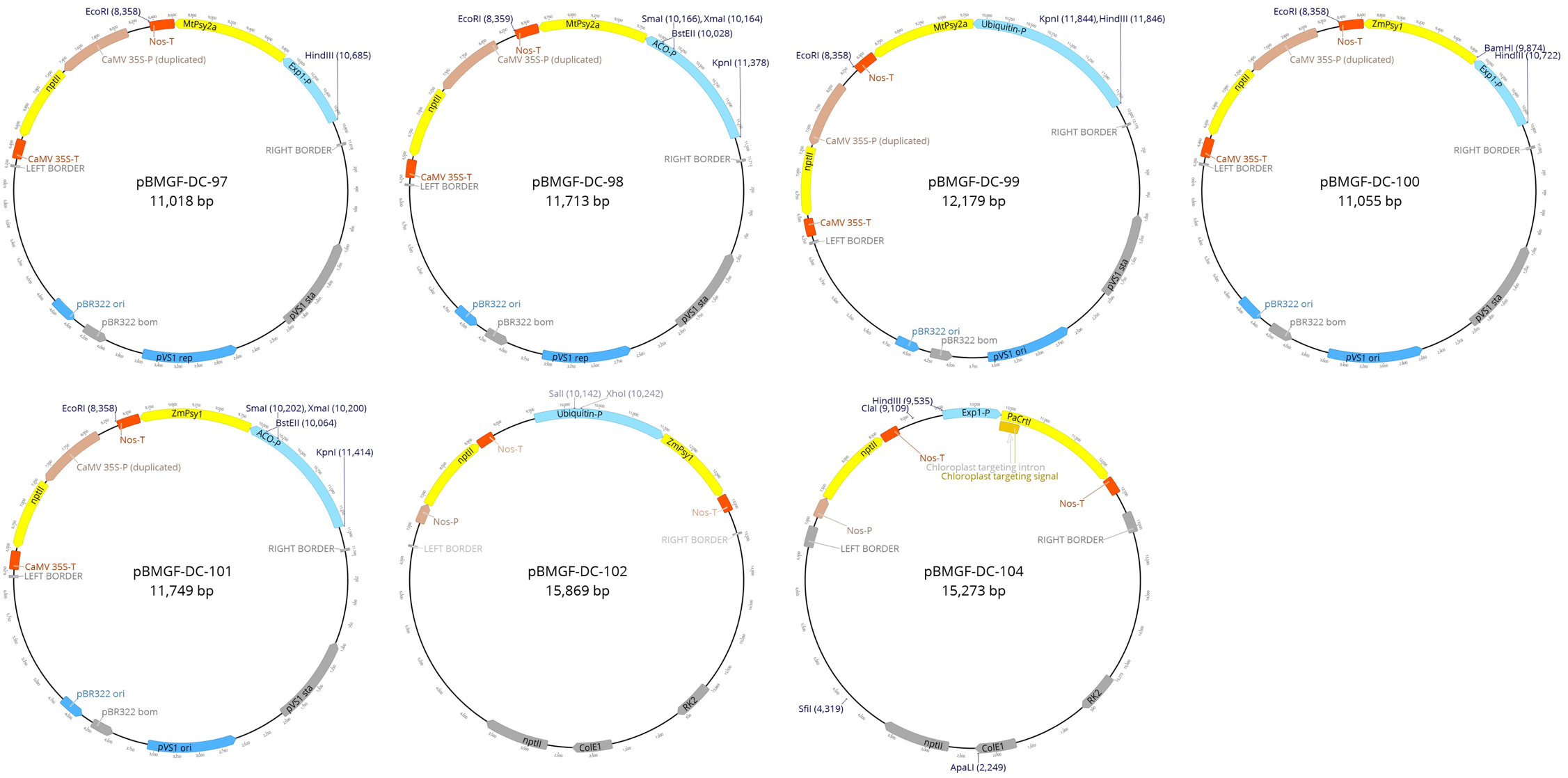


**Figure S1** Schematic representation of binary vectors used for the genetic transformation of banana ECS. Nos, nopaline synthase; CaMV 35S, Cauliflower mosaic virus 35S; ACO, ACC oxidase promoter; Exp1, expansin 1 promoter; Ubi, maize polyubiquitin 1 promoter; *nptII*, neomycin phosphotransferase selectable marker gene; *MtPsy2a*, phytoene synthase 2a gene from the Fe’i banana ‘Asupina’; *ZmPsy1*, phytoene synthase 1 gene from maize (*Zea mays*) and *PaCrtI*, *Pantoea ananatis* phytoene desaturase.

**Table S1** Selected pVA-biofortified ‘Cavendish’ banana lines investigated in this study

| **Line ID** | **Promoter-*transgene*** | **Original plant crop data** | |
| --- | --- | --- | --- |
|  |  | **β-CE (µg/g DW)^** | **Bunch weight (kg)** |
| FT162* | Wild-type | 1.5±0.2 | 40.5 |
| FT166 |  | 1.0±0.1 | 32.5 |
| FT167^#^ |  | NA | 42.5 |
| FT430*^#^ |  | 3.2±0.3 | 33.5 |
| FT448 |  | NA | 31.0 |
| FT187^#^ | Exp1-*ZmPsy1* | 3.5±0.1 | 27.0 |
| FT192^#^ |  | 4.3±0.2 | 17.0 |
| FT201 |  | 2.3±0.0 | 24.0 |
| FT317 |  | 6.6±0.1 | 16.0 |
| FT538 |  | 3.8±0.4 | 25.0 |
| FT467^#^ | ACO-*ZmPsy1* | 7.3±0.3 | 22.0 |
| FT475^#^ |  | 4.3±0.3 | 21.0 |
| FT479^#^ |  | 2.7±0.3 | 22.0 |
| FT483 |  | 1.7±0.3 | 14.0 |
| FT493~ |  | 4.3±0.2 | 21.0 |
| FT588~ |  | 7.7±0.6 | 23.0 |
| FT584^#^ | ACO-*ZmPsy1*+Exp1-*PaCrtI* | 17.1±0.3 | 15.0 |
| FT585^#^ |  | 7.3±0.5 | 25.0 |
| FT587 |  | 11.5±0.7 | 11.0 |
| FT287^#^ | Ubi-*ZmPsy1* | 13.4±1.6 | 18.5 |
| FT309^#^ |  | 11.9±0.5 | 13.0 |
| FT242 | Exp1-*MtPsy2a* | 2.2±0.1 | 22.5 |
| FT246*^#^ |  | 7.3±0.3 | NA |
| FT341^#^ |  | 1.4±0.2 | 32.0 |
| FT342*^#^ |  | 2.8±0.2 | 38.5 |
| FT497^#^ | ACO-*MtPsy2a* | 4.1±0.1 | 26.5 |
| FT504* |  | 16.6±1.1 | 27.0 |
| FT508 |  | 9.2±0.5 | 28.0 |
| FT511* |  | 9.4±1.1 | 27.0 |
| FT518^#^ |  | 15.9±0.7 | 21.0 |
| FT294* | Ubi-*MtPsy2a* | 6.6±0.8 | 26.0 |
| FT295^#^ |  | 5.4±0.5 | 39.0 |
| FT324*^#^ |  | 11.7±1.5 | 22.0 |
| FT328~ |  | 18.7±1.7 | 6.0 |
| FT330 |  | 2.5±0.4 | 40.5 |

Carotenoid content in the mature green fruit of selected lines from the plant crop of FT-1 represent mean β-carotene equivalents (β-CE) ± SD from 3 technical replicates.

^Data previously published in Paul *et al.,* 2017.

*Lines selected for MtPsy2a versus endogene expression studies.

^#^Lines selected for transgene expression studies.

~Lines for which continuous data across three successive generations could not be gathered.

**Table S2** Oligonucleotide primer names and sequences

| **Target gene** | **Primer** | **Sequence (5’ → 3')** | **Amplicon size (bp)** |
| --- | --- | --- | --- |
| *CYP* | qCYP F | GACGGTTCACGCCTCTGTG | 97 |
|  | qCYP R | TGGCTCCTGCTGACGATAATG |  |
| *RPS2* | qRPS2-F | ACTCAACCGTCTTCCCAAAAG | 110 |
|  | qRPS2-R | TCACAATATCAGGCAATCCCG |  |
| *MtPsy2a* | qAPsy2a F | CCAAGGCAGTAATCGTAGGC | 155 |
|  | qNos R | ATGTGATAATCATCGCAAGACC |  |
| *ZmPsy1* | qZmPsy1 F3 | CATTGAGAAATGGCCAGACC | 104 |
|  | qNosPsy1 R3 | AAGACCGGCAACAGGATTC |  |
| *PaCrtI* | qCrtI F2 | CAAAAGCGACAGCAGGTTTG | 159 |
|  | qNos R | ATGTGATAATCATCGCAAGACC |  |


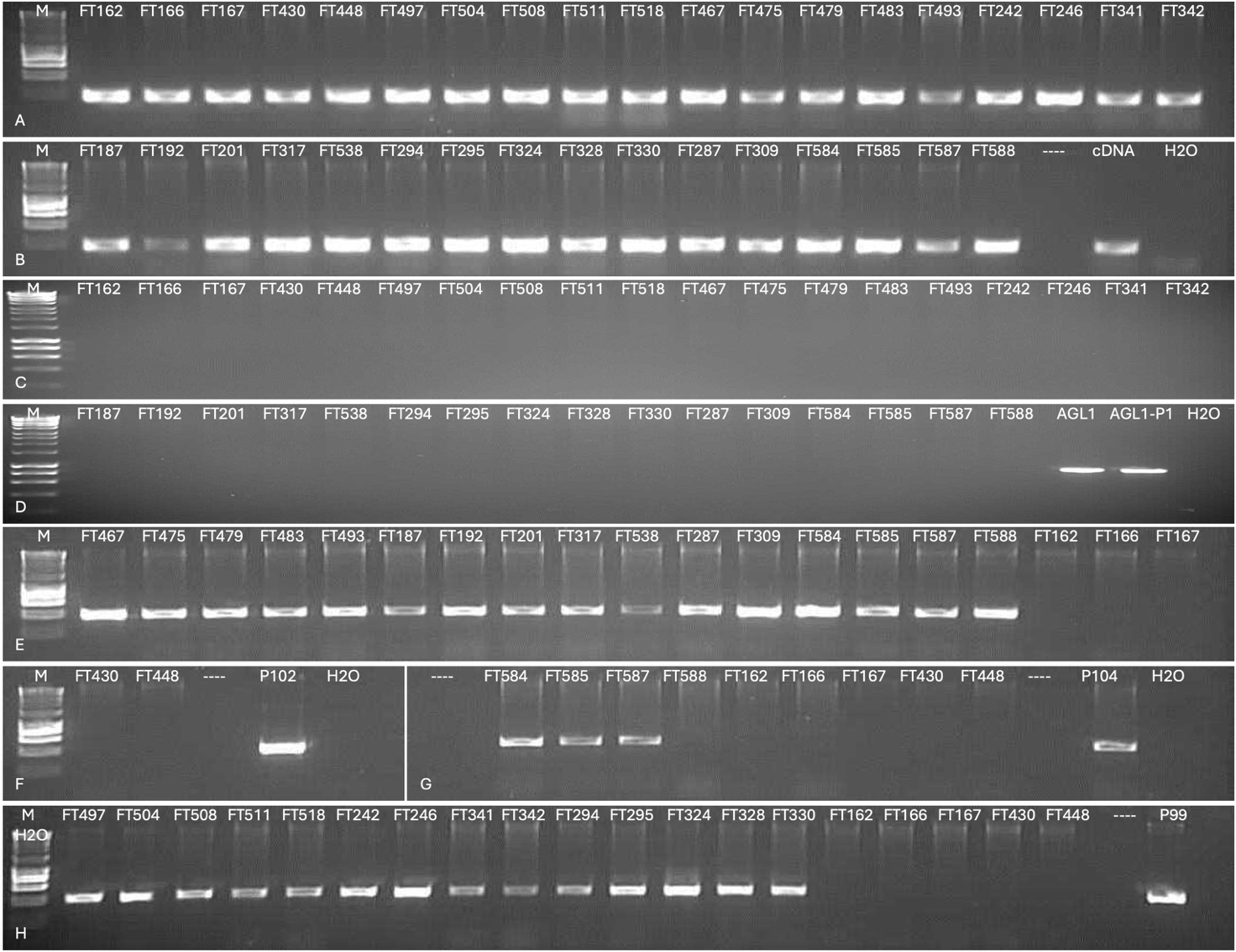


**Figure S2** PCR detection of transgene(s) in selected transgenic ‘Cavendish’ bananas lines. The quality of the extracted genomic DNA was assessed by PCR amplification of the *CYP* housekeeping gene (A and B); detection of residual Agrobacterium contamination by PCR amplification of the *virC* gene from the AGL1 strain of *Agrobacterium tumefaciens* (C and D). The presence of the *ZmPsy1* (E and F), *PaCrtI* (G) and *MtPsy2a* (H) transgenes was detected using transgene specific primer sets. M; HyperLadder 1kb marker; AGL1, wild-type *A. tumefaciens* strain AGL-1; AGL1-P1, pOpt-EBX recombinant AGL-1; cDNA, complementary DNA; H2O, water control; P99, pBMGF-DC-99; P102, pBMGF-DC-102 and P104, pBMGF-DC-104.


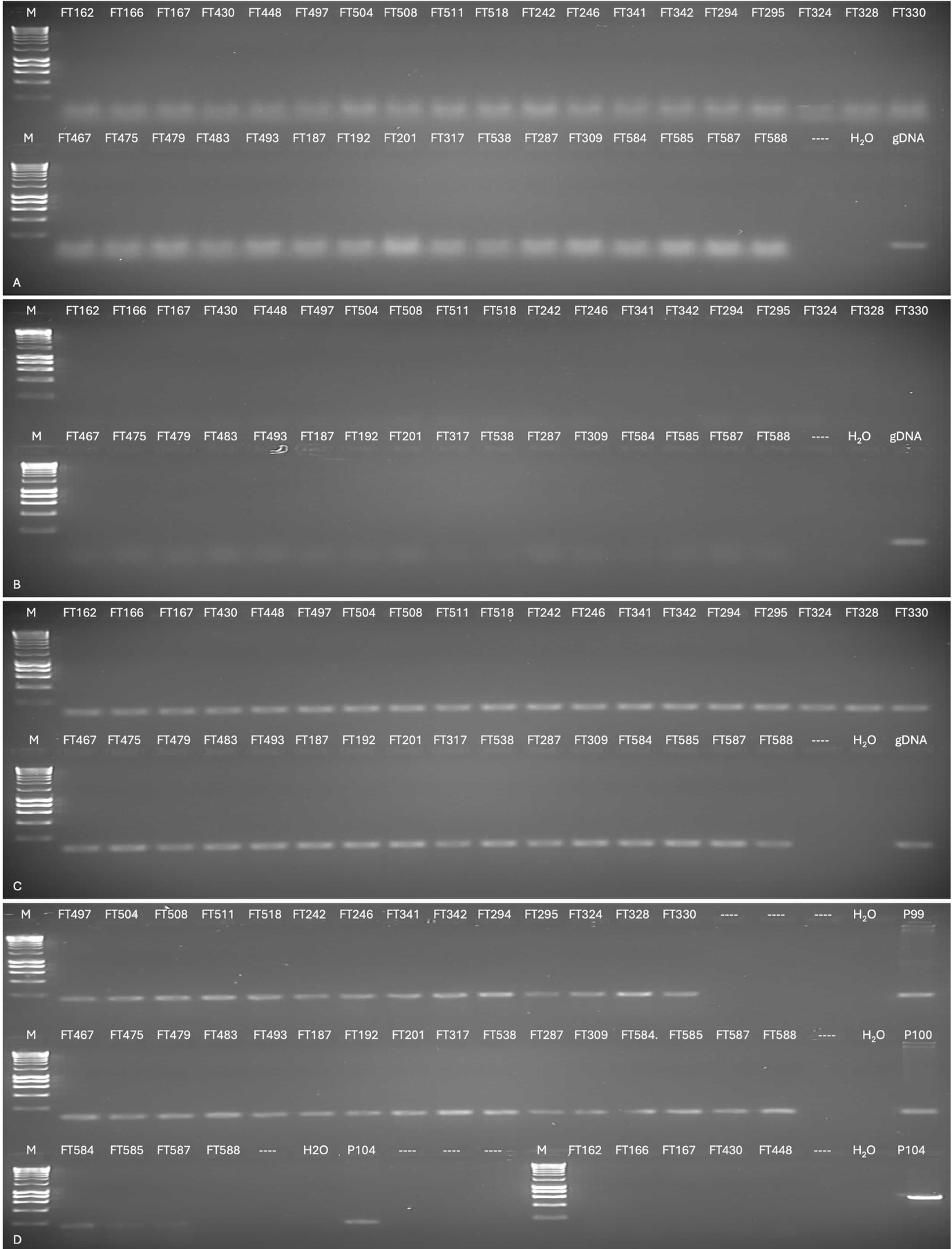


**Figure S3** Detection of transgene mRNA by RT-PCR. DNA contamination was detected by PCR amplification of the *CYP* housekeeping gene in the total RNA extracts (A) and in the DNase treated extracts (B). The quality of cDNA synthesis was assessed by PCR amplification of a *CYP* mRNA transcript (C) followed by detection of transgene specific mRNA (D), *MtPsy2a* (top panel), *ZmPsy1* (middle panel), *PaCrtI* (bottom panel left) and *nptII* (bottom panel right). M; HyperLadder 1kb marker; gDNA, genomic DNA; H2O, water control; P99, pBMGF-DC-99; P100, pBMGF-DC-100 and P104, pBMGF-DC-104.

**Table S3** Number of T-DNA-integrated copies determined by Southern blot analysis

| **Transgene** | **T-DNA copy #** | **Independent line ID** |
| --- | --- | --- |
| *MtPsy2a* | 1 | FT295, FT342, FT497 |
|  | 2 | FT294, FT330, FT511 |
|  | 3 | FT242, FT504 |
|  | 4+ | FT246, FT324, FT328, FT341, FT508, FT518 |
| *ZmPsy1* | 1 | FT317, FT538 |
|  | 2 | FT192 |
|  | 3 | FT287, FT475 |
|  | 4+ | FT187, FT201, FT309, FT467, FT479, FT483, FT493, FT584, FT585, FT587, FT588 |
| *PaCrtI* | 4+ | FT584, FT585, FT587 |

**Table S4** Agronomical characteristics of the sucker crop

| **Line ID** | **Promoter-*transgene*** | **Sucker (S)** | | | | | |
| --- | --- | --- | --- | --- | --- | --- | --- |
|  |  | **Plant height (cm)** | **Plant cycle time (days)** | **Bunch cycle time (days)** | **Bunch weight (kg)** | **Finger number** | **Finger length (cm)** |
| FT162 | Wild-type | 140.3 | 268.8 | 114.3 | 19.5 | 130.8 | 18.6 |
| FT166 |  | 162.5 | 292.3 | 123.0 | 22.3 | 151.0 | 20.3 |
| FT167 |  | 156.0 | 283.3 | 118.0 | 24.6 | 151.0 | 19.9 |
| FT430 |  | 167.0 | 288.3 | 117.8 | 28.1 | 156.0 | 20.4 |
| FT448 |  | 176.3 | 305.8 | 127.8 | 23.1 | 146.3 | 21.0 |
| FT187 | Exp1-*ZmPsy1* | 164.3 | 294.0 | 121.0 | 25.5 | 148.5 | 21.3 |
| FT192 |  | 133.8* | 314.3 | 122.3 | 11.0* | 109.8 | 17.3* |
| FT201 |  | 151.0 | 298.8 | 122.0 | 22.1 | 140.0 | 20.3 |
| FT317 |  | 131.5* | 368.5* | 119.8 | 14.7* | 110.8 | 16.8* |
| FT538 |  | 161.5 | 285.3 | 108.5 | 23.6 | 144.3 | 20.8 |
| FT467 | ACO-*ZmPsy1* | 165.5 | 288.5 | 111.0 | 24.8 | 147.8 | 20.1 |
| FT475 |  | 165.5 | 307.5 | 126.8 | 23.1 | 151.8 | 20.4 |
| FT479 |  | 163.0 | 358.0* | 115.5 | 23.5 | 145.8 | 19.6 |
| FT483 |  | 141.0 | 379.3* | 115.3 | 17.5 | 145.7 | 17.5 |
| FT584 | ACO-*ZmPsy1*+Exp1-*PaCrtI* | 208.5* | 310.8 | 121.5 | 13.9* | 94.3* | 20.6 |
| FT585 |  | 170.0 | 288.0 | 115.8 | 24.1 | 147.0 | 20.0 |
| FT587 |  | 202.7* | 263.0 | 122.0 | 14.7* | 101.0* | 19.5 |
| FT287 | Ubi-*ZmPsy1* | 146.8 | 389.3* | 123.8 | 11.4* | 139.5 | 12.9* |
| FT309 |  | 123.0* | 543.3* | 111.5 | 6.9* | 67.3* | 16.1* |
| FT242 | Exp1-*MtPsy2a* | 152.0 | 285.0 | 113.8 | 21.8 | 144.8 | 18.8 |
| FT246 |  | 139.8 | 290.3 | 112.5 | 13.2* | 110.3 | 16.9* |
| FT341 |  | 165.8 | 291.5 | 124.0 | 26.4 | 157.3 | 19.8 |
| FT342 |  | 157.5 | 265.3 | 111.3 | 26.1 | 151.0 | 20.7 |
| FT497 | ACO-*MtPsy2a* | 152.3 | 282.5 | 121.5 | 21.5 | 133.0 | 18.4 |
| FT504 |  | 159.3 | 298.5 | 127.0 | 21.6 | 147.0 | 18.7 |
| FT508 |  | 177.5 | 307.8 | 127.5 | 27.4 | 152.8 | 21.4 |
| FT511 |  | 172.3 | 300.8 | 116.0 | 26.5 | 148.8 | 21.1 |
| FT518 |  | 159.3 | 310.8 | 116.3 | 17.6 | 144.0 | 19.5 |
| FT294 | Ubi-*MtPsy2a* | 147.8 | 294.0 | 114.0 | 19.8 | 119.5 | 18.3 |
| FT295 |  | 163.3 | 290.0 | 116.8 | 26.1 | 135.3 | 20.4 |
| FT324 |  | 131.0* | 368.3* | 130.0 | 12.4* | 100.3* | 18.4 |
| FT330 |  | 282.8* | 298.5 | 115.0 | 22.0 | 108.8* | 20.6 |

* Indicates values that are statistically different from the average of the control wild-type data at 95% confidence (ANOVA and Tukeys HSD post-hoc test).

Data are average of 4 biological replicates (n=4) except for FT483 (n=3).

**Table S5** Agronomical characteristics of the sucker ratoon 1 crop

| **Line ID** | **Promoter-*transgene*** | **Sucker ratoon 1 (SR1)** | | | | | |
| --- | --- | --- | --- | --- | --- | --- | --- |
|  |  | **Plant height (cm)** | **Plant cycle time (days)** | **Bunch cycle time (days)** | **Bunch weight (kg)** | **Finger number** | **Finger length (cm)** |
| FT162 | Wild-type | 183.3 | 267.5 | 93.0 | 28.9 | 188.0 | 19.8 |
| FT166 |  | 195.3 | 258.5 | 98.3 | 32.5 | 202.5 | 20.3 |
| FT167 |  | 204.3 | 242.5 | 94.8 | 31.3 | 193.5 | 20.3 |
| FT430 |  | 205.0 | 255.8 | 93.0 | 33.8 | 193.3 | 21.1 |
| FT448 |  | 232.8 | 242.8 | 99.3 | 30.8 | 205.3 | 19.9 |
| FT187 | Exp1-*ZmPsy1* | 201.3 | 242.3 | 90.3 | 32.3 | 191.8 | 21.4 |
| FT192 |  | 137.0* | 296.5 | 107.5 | 9.0* | 107.0* | 16.9* |
| FT201 |  | 182.5 | 241.0 | 89.8 | 28.1 | 166.8 | 20.9 |
| FT317 |  | 141.8* | 290.8 | 124.5 | 12.9* | 101.5* | 18.4 |
| FT538 |  | 195.8 | 230.3 | 96.8 | 33.9 | 202.5 | 21.0 |
| FT467 | ACO-*ZmPsy1* | 202.5 | 284.8 | 98.0 | 28.9 | 187.3 | 20.4 |
| FT475 |  | 193.5 | 257.0 | 98.3 | 30.9 | 205.3 | 20.3 |
| FT479 |  | 196.3 | 295.5 | 112.7 | 25.5* | 164.3 | 19.5 |
| FT483 |  | 165.3 | 329.0 | 124.3 | 23.0 | 182.3 | 15.0* |
| FT584 | ACO-*ZmPsy1*+Exp1-*PaCrtI* | 301.7* | 199.5 | 96.3 | 22.5* | 150.8 | 20.3 |
| FT585 |  | 202.3 | 244.8 | 94.5 | 32.6 | 185.0 | 20.5 |
| FT587 |  | 275.5* | 224.0 | 81.0* | 13.8* | 131.8* | 18.3 |
| FT287 | Ubi-*ZmPsy1* | 163* | 322.5 | 117.3 | 13.1* | 169.0 | 12.8* |
| FT309 |  | 127* | 541.3* | 104.0 | 2.9* | 85.0* | 11.5* |
| FT242 | Exp1-*MtPsy2a* | 184.8 | 262.0 | 94.5 | 24.1 | 150.0 | 20.1 |
| FT246 |  | 143.3* | 239.0 | 94.8 | 12.3* | 106.0* | 16.1* |
| FT341 |  | 208.0 | 239.8 | 98.5 | 34.6 | 213.5 | 21.0 |
| FT342 |  | 188.3 | 297.3 | 99.5 | 29.5 | 166.5 | 21.0 |
| FT497 | ACO-*MtPsy2a* | 182.8 | 264.5 | 98.8 | 30.3 | 183.8 | 20.8 |
| FT504 |  | 170.8 | 231.8 | 98.5 | 22.5 | 149.8 | 18.8 |
| FT508 |  | 197.5 | 257.0 | 102.8 | 31.4 | 180.0 | 19.6 |
| FT511 |  | 193.8 | 244.5 | 92.8 | 33.6 | 175.0 | 19.9 |
| FT518 |  | 168.0* | 231.3 | 97.0 | 22.4 | 175.0 | 19.1 |
| FT294 | Ubi-*MtPsy2a* | 164.8* | 262.0 | 96.5 | 21.3 | 139.8 | 20.0 |
| FT295 |  | 201.5 | 274.3 | 100.5 | 30.6 | 180.8 | 20.4 |
| FT324 |  | 158.0* | 283.5 | 115.8 | 16.8* | 125.5* | 20.0 |
| FT330 |  | 375.3* | 223.8 | 81.7 | 21.0* | 124.3* | 20.7 |

* Indicates values that are statistically different from the average of the control wild-type data at 95% confidence (ANOVA and Tukeys HSD post-hoc test).

Data are average of 4 biological replicates (n=4) except for FT483 (n=3).

**Table S6** Agronomical characteristics of the sucker ratoon 2 crop

| **Line ID** | **Promoter-*transgene*** | **Sucker ratoon 2 (SR2)** | | | | | |
| --- | --- | --- | --- | --- | --- | --- | --- |
|  |  | **Plant height (cm)** | **Plant cycle time (days)** | **Bunch cycle time (days)** | **Bunch weight (kg)** | **Finger number** | **Finger length (cm)** |
| FT162 | Wild-type | 177.3 | 275.0 | 108.8 | 27.9 | 170.5 | 21.3 |
| FT166 |  | 200.3 | 311.0 | 98.0 | 35.6 | 196.0 | 22.1 |
| FT167 |  | 185.3 | 278.3 | 117.0 | 30.1 | 174.3 | 22.4 |
| FT430 |  | 202.5 | 295.0 | 100.5 | 36.3 | 194.3 | 23.0 |
| FT448 |  | 198.0 | 289.8 | 108.8 | 35.7 | 211.5 | 21.8 |
| FT187 | Exp1-*ZmPsy1* | 181.3 | 299.3 | 108.3 | 31.7 | 167.0 | 23.4 |
| FT192 |  | 146.0 | 332.0 | 314.3* | 10.6* | 113.0* | 15.5* |
| FT201 |  | 175.0 | 309.8 | 103.8 | 29.1 | 169.5 | 21.8 |
| FT317 |  | 154.3 | 293.8 | 99.8 | 15.2* | 113.8* | 17.6* |
| FT538 |  | 193.8 | 297.5 | 106.3 | 32.1 | 191.0 | 21.1 |
| FT467 | ACO-*ZmPsy1* | 207.8 | 278.3 | 97.3 | 33.7 | 185.5 | 21.3 |
| FT475 |  | 209.0 | 327.3 | 98.3 | 29.9 | 188.3 | 19.9 |
| FT479 |  | 176.5* | 529.5 | 95.0 | 15.3* | 105.5* | 17.8 |
| FT483 |  | 177.7 | 326.7 | 107.0 | 23.6 | 182.0 | 17* |
| FT584 | ACO-*ZmPsy1*+Exp1-*PaCrtI* | 340.8* | 227.0 | 110.0 | 16.0* | 120.5* | 20.7 |
| FT585 |  | 194.0 | 285.8 | 103.5 | 33.3 | 187.5 | 23.0 |
| FT587 |  | 316.5* | 276.0 | 116.5 | 16.8* | 125.3 | 20.4 |
| FT287 | Ubi-*ZmPsy1* | 190.7 | 363.3 | 117.0 | 14.5* | 172.0 | 12.9* |
| FT309 |  | NA | NA | NA | NA | NA | NA |
| FT242 | Exp1-*MtPsy2a* | 187.5 | 318.5 | 92.3 | 26.5 | 141.8 | 22.5 |
| FT246 |  | 131.5* | 292.3 | 112.3 | 9.3* | 91.3* | 15.1* |
| FT341 |  | 206.8 | 272.8 | 109.0 | 36.6 | 196.5 | 23.1 |
| FT342 |  | 206.5 | 267.8 | 113.5 | 36.7 | 216.0 | 22.8 |
| FT497 | ACO-*MtPsy2a* | 175.0 | 288.5 | 99.3 | 28.1 | 165.8 | 22.1 |
| FT504 |  | 166.0 | 272.0 | 115.3 | 19.1* | 127.8 | 21.0 |
| FT508 |  | 196.8 | 273.0 | 98.0 | 31.8 | 173.5 | 22.0 |
| FT511 |  | 194.8 | 274.8 | 103.3 | 35.4 | 190.5 | 22.5 |
| FT518 |  | 157.3 | 316.3 | 104.8 | 19.8* | 128.3 | 20.4 |
| FT294 | Ubi-*MtPsy2a* | 164.3 | 290.0 | 100.5 | 20.2 | 130.8 | 20.6 |
| FT295 |  | 196.3 | 269.8 | 96.5 | 29.8 | 181.8 | 22.4 |
| FT324 |  | 161.8 | 355.3 | 113.5 | 15.8* | 96.0* | 18.1* |
| FT330 |  | 382.5* | 269.0 | 90.5 | 24.9* | 132.2 | 21.3 |

* Indicates values that are statistically different from the average of the control wild-type data at 95% confidence (ANOVA and Tukeys HSD post-hoc test).

Data are average of 4 biological replicates (n=4) except for FT483 (n=3).

NA, data not available.

**Table S7** Number of fruit samples collected and analysed by HPLC across three generations

| **Promoter-*gene*** | **Sucker (S)** | | **Sucker ratoon 1 (SR1)** | | **Sucker ratoon 2 (SR2)** | |
| --- | --- | --- | --- | --- | --- | --- |
|  | **Green** | **Ripe** | **Green** | **Ripe** | **Green** | **Ripe** |
| Wild-type | 20 | 20 | 19 | 20 | 19 | 19 |
| Exp1-*ZmPsy1* | 18 | 18 | 20 | 20 | 19 | 19 |
| Exp1-*MtPsy2a* | 16 | 16 | 16 | 16 | 16 | 16 |
| ACO-*ZmPsy1* | 15 | 16 | 14 | 14 | 13 | 13 |
| ACO-*MtPsy2a* | 20 | 20 | 19 | 19 | 20 | 20 |
| Ubi-*ZmPsy1* | 8 | 8 | 5 | 5 | 6 | 6 |
| Ubi-*MtPsy2a* | 16 | 16 | 15 | 15 | 15 | 15 |
| ACO-*ZmPsy1+*Exp1*-PaCrtI* | 12 | 12 | 8 | 8 | 11 | 11 |
| Total | 125 | 126 | 116 | 117 | 119 | 119 |

**Table S8** Carotenoid content in the mature green fruit of transgenic Cavendish banana lines across four generations

| **Lines** | **Promoter-*transgene*** | **β-carotene equivalents (µg/g DW)** | | | |  |
| --- | --- | --- | --- | --- | --- | --- |
|  |  |  |  |  |  |  |
|  |  | **P**^ | **S** | **SR1** | **SR2** |  |
| FT162 | Wild-Type | 1.46 ± 0.19 | 4.65 ± 0.46a | 1.57 ± 0.34b | 0.98 ± 0.12c |  |
| FT166 |  | 0.98 ± 0.05 | 6.61 ± 2.05a | 1.43 ± 0.29b | 2.76 ± 0.75c |  |
| FT167 |  | N/A | 4.11 ± 0.53a | 1.32 ± 0.32b | 2.51 ± 0.43c |  |
| FT430 |  | 3.23 ± 0.33 | 6.46 ± 1.37a | 2.95 ± 0.24b | 1.16 ± 0.22c |  |
| FT448 |  | N/A | 7.96 ± 0.35a | 1.55 ± 0.45b | 2.18 ± 0.48c |  |
| FT187 | Exp1-*ZmPsy1* | 3.53 ± 0.06 | 7.03 ± 0.79a | 2.86 ± 1.26b | 3.54 ± 1.08b |  |
| FT192 |  | 4.27 ± 0.18 | 20.34 ± 4.75a | 9.69 ± 1.45b | 13.15 ± 1.87c |  |
| FT201 |  | 2.25 ± 0.03 | 9.12 ± 3.26a | 6.99 ± 1.75a | 4.48 ± 1.78b |  |
| FT317 |  | 6.56 ± 0.08 | 11.43 ± 9.25a | 10.02 ± 0.99a | 10.50 ± 2.02a |  |
| FT538 |  | 3.77 ± 0.40 | 10.94 ± 3.30a | 2.02 ± 0.69b | 2.73 ± 0.69b |  |
| FT467 | ACO-*ZmPsy1* | 7.26 ± 0.29 | 10.45 ± 1.83a | 4.91 ± 2.08b | 5.10 ± 1.48b |  |
| FT475 |  | 4.33 ± 0.28 | 20.69 ± 2.25a | 4.11 ± 1.17b | 8.22 ± 2.85c |  |
| FT479 |  | 2.68 ± 0.30 | 20.52 ± 10.92a | 15.00 ± 5.76a | 20.76 ± 5.32a |  |
| FT483 |  | 1.69 ± 0.26 | 18.80 ± 6.63a | 7.56 ± 3.17b | 8.15 ± 4.29b |  |
| FT584 | ACO-*ZmPsy1* + Exp1-*PaCrtI* | 17.14 ± 0.32 | 27.00 ± 11.50a | 7.38 ± 0.89b | 27.92 ± 0.62a |  |
| FT585 |  | 7.29 ± 0.49 | 12.25 ± 2.79a | 3.35 ± 0.54b | 2.46 ± 1.06b |  |
| FT587 |  | 11.52 ± 0.71 | 21.50 ± 3.87a | 6.93 ± 0.30b | 12.68 ± 7.30b |  |
| FT287 | Ubi-*ZmPsy1* | 13.40 ± 1.64 | 39.71 ± 0.96a | 45.86 ± 15.82a | 46.13 ± 21.30a |  |
| FT309 |  | 11.86 ± 0.53 | 46.92 ± 11.13a | 32.66 ± 0.52b | 17.61 ± 2.03c |  |
| FT242 | Exp1-*MtPsy2a* | 2.23 ± 0.09 | 8.62 ± 1.38a | 2.36 ± 0.53b | 4.53 ± 1.37c |  |
| FT246 |  | 7.28 ± 0.32 | 18.17 ± 3.99a | 10.45 ± 1.35b | 6.38 ± 2.55c |  |
| FT341 |  | 1.39 ± 0.20 | 9.27 ± 2.42a | 2.63 ± 0.98b | 1.07 ± 0.25c |  |
| FT342 |  | 2.83 ± 0.20 | 10.57 ± 1.63a | 5.87 ± 3.89b | 10.27 ± 4.97a |  |
| FT497 | ACO-*MtP*sy2a | 4.14 ± 0.15 | 13.44 ± 4.39a | 5.03 ± 0.91b | 4.65 ± 0.33b |  |
| FT504 |  | 16.55 ± 1.12 | 20.01 ± 2.86a | 17.44 ± 6.74a | 9.44 ± 6.70b |  |
| FT508 |  | 9.17 ± 0.54 | 15.65 ± 1.18a | 5.85 ± 1.39b | 5.74 ± 0.82b |  |
| FT511 |  | 9.40 ± 1.12 | 14.42 ± 1.70a | 4.36 ± 1.14b | 4.72 ± 2.58b |  |
| FT518 |  | 15.87 ± 0.67 | 23.08 ± 2.92a | 25.72 ± 3.28b | 10.27 ± 1.14c |  |
| FT294 | Ubi-*MtPsy2a* | 6.56 ± 0.81 | 29.03 ± 2.04a | 12.87 ± 2.79b | 17.21 ± 1.41b |  |
| FT295 |  | 5.39 ± 0.55 | 10.52 ± 1.83a | 5.89 ± 0.81b | 6.87 ± 1.74b |  |
| FT324 |  | 11.68 ± 1.45 | 54.99 ± 12.81a | 37.25 ± 6.48b | 75.15 ± 7.51a |  |
| FT330 |  | 2.49 ± 0.40 | 11.91 ± 3.29a | 3.96 ± 0.48b | 6.96 ± 5.45b |  |

β-CE, β-carotene equivalents. NA, data not available.

Data presented for the P crop are mean β-carotene equivalents (β-CE) ± SD from 3 technical replicates and therefore were not used in statistical analysis.

Data for the S, SR1 and SR2 crops represent mean β-CE ± SD from 4 plants (biological replicates) with 3 technical replicates (n=12).

S, sucker crop; SR1, sucker ratoon 1 crop; SR2, sucker ratoon 2 crop; NA, data not available.

Statistical analysis compared means of each line between all three crops (S, SR1 and SR2). Values with the same letters are not statistically different from each other at 95% confidence (ANOVA and Tukey’s HSD post-hoc test).

^Data previously published in Paul *et al.,* 2017.


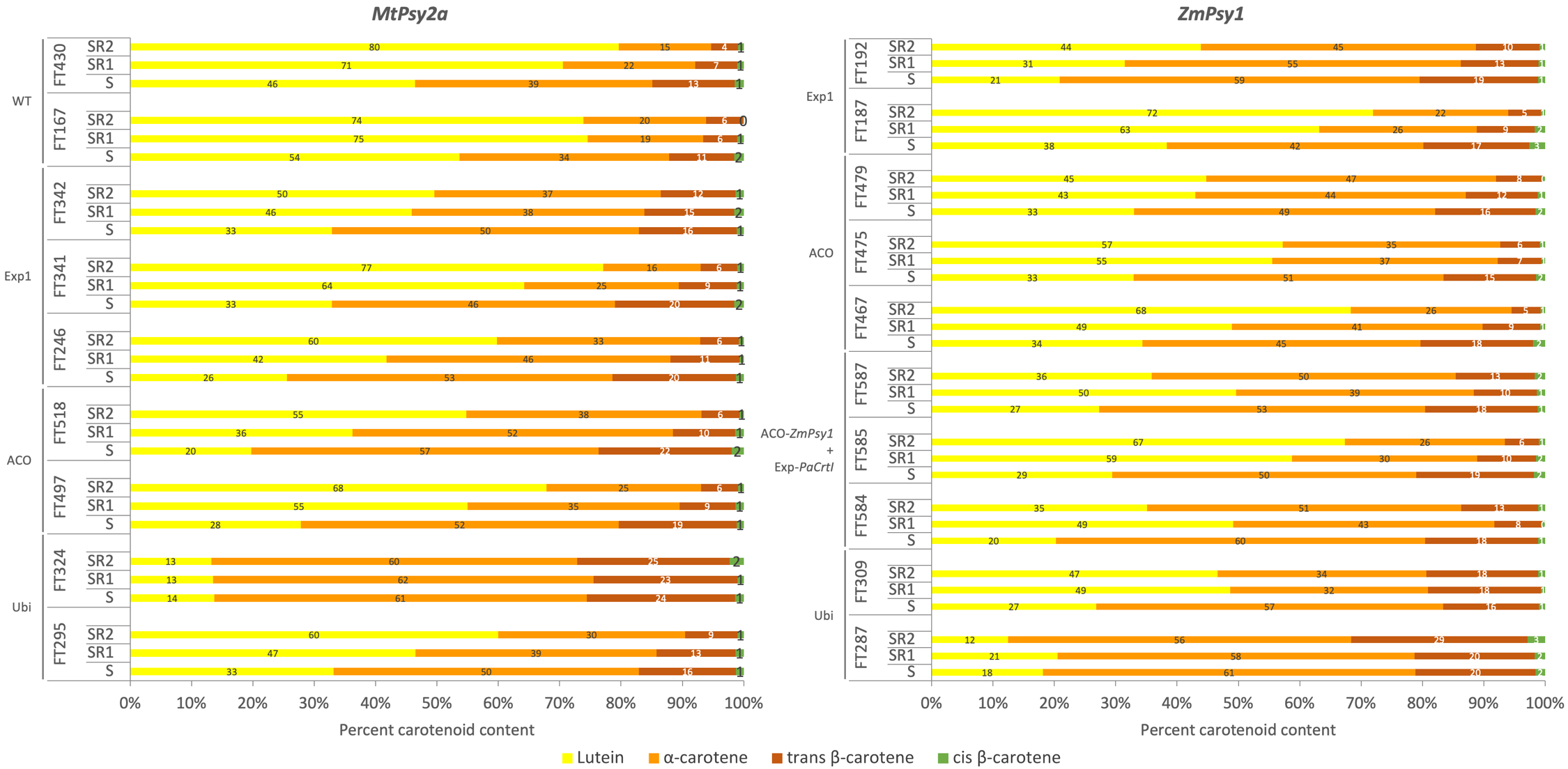


**Figure S4** Percentage carotenoid composition in the green fruit of wild-type and transgenic ‘Cavendish’ banana lines across 3 successive generations. Lines FT167 and FT430 are wild-type while transgenic lines FT246, FT341 and FT342 (Exp1-*MtPsy2a*); FT497 and FT518 (ACO-*MtPsy2a*); FT295 and FT324 (Ubi-*MtPsy2a*); FT187 and FT192 (Exp1-*ZmPsy1*); FT467, FT475 and FT479 (ACO-*ZmPsy1*); FT584, FT585 and FT587 (ACO-*ZmPsy1*+Exp1-*PaCrtI*); FT287 and FT309 (Ubi-*ZmPsy1*). S, sucker crop; SR1, sucker ratoon 1 crop and SR2, sucker ratoon 2 crop. Data presented is an average of 3 technical replicates for 4 biological replicates (n=12) expressed as percentages.

**Table S9** Carotenoid content in the full ripe fruit of transgenic Cavendish banana lines across four generations

| **Lines** | **Promoter-*transgene*** | **β-carotene equivalents (µg/g DW)** | | | |  |  |
| --- | --- | --- | --- | --- | --- | --- | --- |
|  |  |  |  |  |  |  |  |
|  |  | **P**^ | **S** | **SR1** | **SR2** |  | |
| FT162 | Wild-Type | 1.63 ± 0.08 | 6.01 ± 1.57a | 2.13 ± 0.22b | 1.04 ± 0.24c |  | |
| FT166 |  | 1.40 ± 0.14 | 7.28 ± 1.93a | 1.87 ± 0.84b | 2.90 ± 0.55b |  | |
| FT167 |  | N/A | 6.14 ± 1.57a | 1.89 ± 0.29b | 2.50 ± 1.33b |  | |
| FT430 |  | 2.80 ± 0.36 | 8.34 ± 2.92a | 1.66 ± 0.31b | 3.32 ± 0.81b |  | |
| FT448 |  | N/A | 9.44 ± 1.38a | 2.13 ± 0.35b | 2.46 ± 0.40b |  | |
| FT187 | Exp1-*ZmPsy1* | 5.33 ± 0.11 | 11.03 ± 1.28a | 3.51 ± 1.24b | 3.51 ± 0.28b |  | |
| FT192 |  | 6.44 ± 0.20 | 31.19 ± 4.53a | 12.27 ± 1.99b | 12.57 ± 2.64b |  | |
| FT201 |  | 3.15 ± 0.30 | 14.97 ± 5.41a | 3.56 ± 0.60b | 6.09 ± 2.14b |  | |
| FT317 |  | 7.85 ± 0.53 | 15.56 ± 10.96a | 12.87 ± 2.43a | 11.47 ± 1.50a |  | |
| FT538 |  | 9.36 ± 0.91 | 14.84 ± 4.61a | 2.53 ± 0.38b | 3.43 ± 0.43b |  | |
| FT467 | ACO-*ZmPsy1* | 10.35 ± 1.14 | 13.39 ± 2.63a | 5.25 ± 1.54b | 5.70 ± 0.82b |  | |
| FT475 |  | 10.52 ± 0.94 | 22.04 ± 4.04a | 4.04 ± 0.89b | 8.26 ± 3.74c |  | |
| FT479 |  | 13.59 ± 0.74 | 16.23 ± 8.21ab | 13.82 ± 3.92a | 23.19 ± 10.63b |  | |
| FT483 |  | 9.11 ± 0.78 | 16.89 ± 6.76a | 8.77 ± 1.39b | 8.29 ± 2.52b |  | |
| FT584 | ACO-*ZmPsy1* + Exp1-*PaCrtI* | 11.16 ± 0.70 | 32.77 ± 10.18a | 8.03 ± 1.49b | 27.83 ± 0.73a |  | |
| FT585 |  | 5.59 ± 0.44 | 11.48 ± 2.50a | 3.89 ± 0.72b | 2.91 ± 0.51b |  | |
| FT587 |  | N/A | 22.21 ± 5.12a | 7.25 ± 0.09b | 13.44 ± 4.35b |  | |
| FT287 | Ubi-*ZmPsy1* | 15.84 ± 0.88 | 60.95 ± 7.43a | 67.46 ± 18.69a | 32.58 ± 9.82b |  | |
| FT309 |  | 14.71 ± 1.05 | 18.46 ± 6.78a | 33.34 ± 0.77b | 33.62 ± 11.79b |  | |
| FT242 | Exp1-*MtPsy2a* | 3.69 ± 0.26 | 7.99 ± 0.93a | 3.84 ± 0.67b | 4.34 ± 0.72b |  | |
| FT246 |  | 9.34 ± 0.45 | 19.63 ± 4.64a | 13.23 ± 1.30b | 7.80 ± 2.66c |  | |
| FT341 |  | 3.14 ± 0.21 | 8.10 ± 1.95a | 3.37 ± 0.69b | 1.42 ± 0.19c |  | |
| FT342 |  | 4.24 ± 0.14 | 8.98 ± 1.22a | 7.87 ± 4.97a | 11.41 ± 4.55a |  | |
| FT497 | ACO-*MtP*sy2a | 10.45 ± 2.39 | 12.87 ± 3.79a | 4.71 ± 0.77b | 5.99 ± 1.39b |  | |
| FT504 |  | 11.98 ± 0.44 | 24.66 ± 1.59a | 19.24 ± 8.07b | 9.78 ± 5.38c |  | |
| FT508 |  | 7.08 ± 0.37 | 19.01 ± 4.86a | 5.62 ± 1.19b | 7.02 ± 3.15b |  | |
| FT511 |  | 7.33 ± 1.19 | 13.58 ± 1.70a | 5.02 ± 1.31b | 5.17 ± 2.49b |  | |
| FT518 |  | 10.73 ± 0.90 | 35.90 ± 6.25a | 11.24 ± 1.30b | 13.42 ± 2.25b |  | |
| FT294 | Ubi-*MtPsy2a* | 9.66 ± 0.87 | 25.18 ± 2.48a | 11.36 ± 1.26b | 14.32 ± 4.30c |  | |
| FT295 |  | 6.06 ± 1.15 | 11.99 ± 2.38a | 7.72 ± 1.77b | 7.70 ± 1.27b |  | |
| FT324 |  | 16.10 ± 1.11 | 50.13 ± 16.59ab | 38.85 ± 9.67a | 64.16 ± 5.54b |  | |
| FT330 |  | 4.93 ± 0.26 | 15.05 ± 6.08a | 4.53 ± 0.66b | 8.97 ± 4.88b |  | |

β-CE, β-carotene equivalents. NA, data not available.

Data presented for the P crop are mean β-carotene equivalents (β-CE) ± SD from 3 technical replicates and therefore were not used in statistical analysis.

Data for the S, SR1 and SR2 crops represent mean β-CE ± SD from 4 plants (biological replicates) with 3 technical replicates (n=12).

S, sucker crop; SR1, sucker ratoon 1 crop; SR2, sucker ratoon 2 crop; NA, data not available.

Statistical analysis compared means of each line between all three crops (S, SR1 and SR2). Values with the same letters are not statistically different from each other at 95% confidence (ANOVA and Tukey’s HSD post-hoc test).

^Data previously published in Paul *et al.,* 2017.


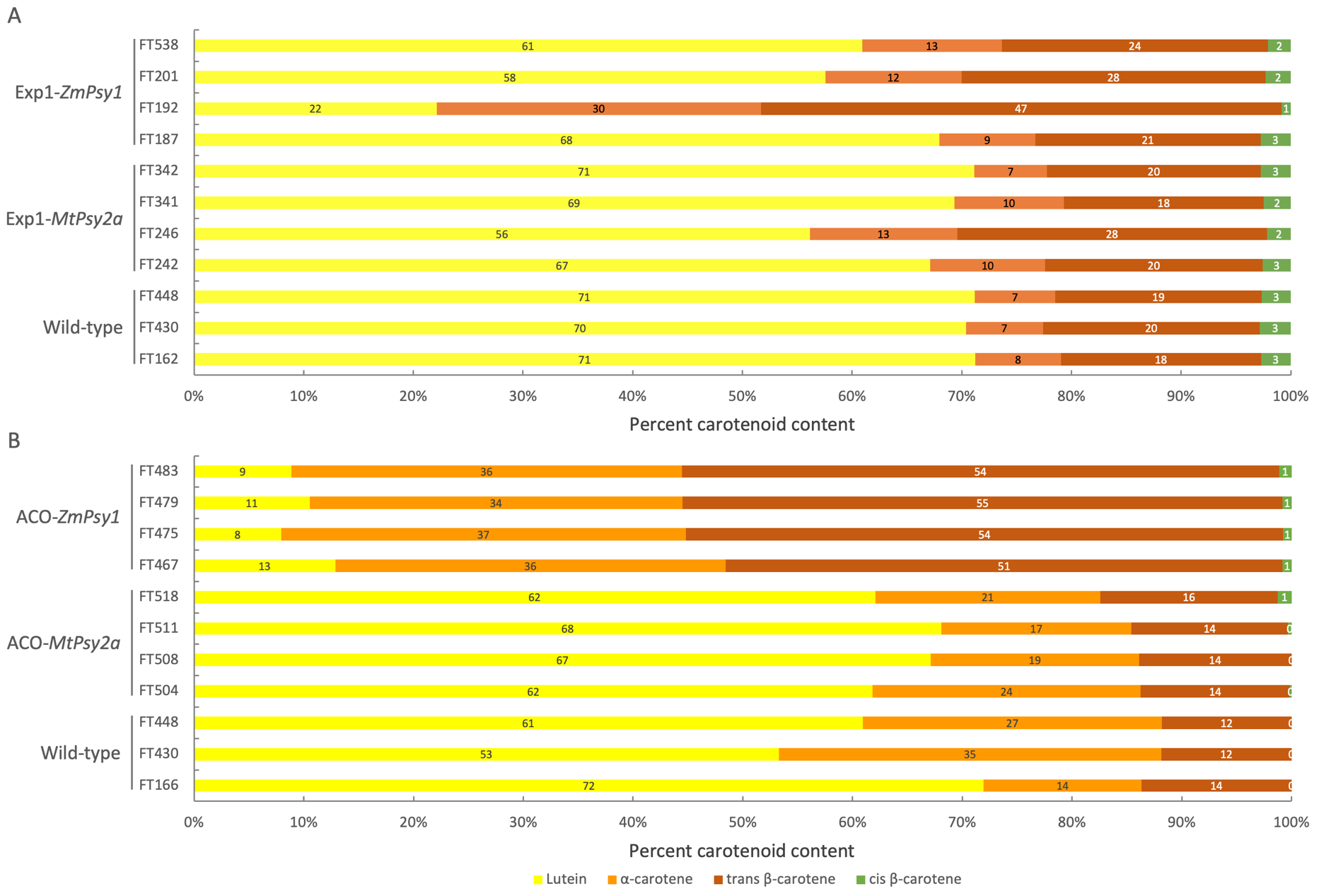


**Figure S5** Carotenoid composition in the leaves and peel of wild-type and transgenic ‘Cavendish’ banana lines with a ‘golden’ phenotype. Carotenoid content in banana leaf (A) and peel from mature unripe fruit (B) was measured by HPLC in samples collected from the sucker crop and the data presented is an average from 4 biological replicates (with 3 technical replicates n=12) expressed as percentage. Lines presented are: wild-type lines FT162, FT430 and FT448; Exp1-*ZmPsy1* ‘golden’ leaf lines FT187, FT192, FT201 and FT538; Exp1-*MtPsy2a* lines FT242, FT246, FT341 and FT34; ACO-*ZmPsy1* ‘golden’ peel lines FT467, FT475, FT479 and FT483 and ACO-*MtPsy2a* lines FT504, FT508, FT511 and FT518.

**Table S10** Environmental conditions influencing green mature fruit carotenoid accumulation and bunch filling time in wild-type and transgenic ‘Cavendish’ banana

| **Months** | **Jan** | **Feb** | **Mar** | **Apr** | **May** | **Jun** | **Jul** | **Aug** | **Sep** | **Oct** | **Nov** | **Dec** |
| --- | --- | --- | --- | --- | --- | --- | --- | --- | --- | --- | --- | --- |
| **Environmental conditions** | | | | | | | | | | | | |
| Temperature (ᵒC) | 32 | 32 | 30 | 29 | 27 | 25 | 24 | 25 | 28 | 29 | 31 | 31 |
| Rainfall (mm) | 429 | 410 | 624 | 320 | 346 | 205 | 159 | 101 | 60 | 78 | 79 | 174 |
| Solar exposure (MJ/m²) | 22.3 | 20.1 | 17.1 | 17.0 | 14.6 | 14.1 | 15.1 | 18.1 | 21.1 | 23.2 | 23.6 | 23.4 |
| **Wild-type** | | | | | | | | | | | | |
| β-CE (µg/g DW) | 1.6 | 2.2 | 1.7 | 1.7 | N/A | 4.6 | 6.0 | 8.2 | N/A | N/A | 2.0 | 1.7 |
| Bunch filling time (days) | 105 | 98 | 94 | 98 | N/A | 111 | 122 | 132 | N/A | N/A | 123 | 112 |
| **Exp1-*MtPsy2a*** | | | | | | | | | | | | |
| β-CE (µg/g DW) | 3.6 | 5.8 | 5.2 | 2.2 | 17.7 | 10.6 | 11.7 | 11.6 | N/A | N/A | 5.3 | 4.5 |
| Bunch filling time (days) | 103 | 92 | 94 | 97 | 101 | 111 | 117 | 127 | N/A | N/A | 116 | 113 |
| **Exp1-*ZmPsy1*** | | | | | | | | | | | | |
| β-CE (µg/g DW) | 3.8 | 4.4 | 4.9 | 7.2 | 10.0 | 9.4 | 11.2 | 16.7 | 10.8 | 3.4 | 6.7 | 3.2 |
| Bunch filling time (days) | 101 | 91 | 91 | 99 | 108 | 108 | 118 | 130 | 139 | 125 | 126 | 113 |
| **ACO-*MtPsy2a*** | | | | | | | | | | | | |
| β-CE (µg/g DW) | 8.6 | 12.8 | 11.8 | 5.1 | 4.8 | 9.6 | 17.8 | 17.5 | N/A | N/A | 13.9 | 5.6 |
| Bunch filling time (days) | 96 | 99 | 97 | 100 | 103 | 117 | 119 | 130 | N/A | N/A | 137 | 103 |
| **ACO-*ZmPsy1*** | | | | | | | | | | | | |
| β-CE (µg/g DW) | 7.6 | 7.4 | 10.2 | 4.2 | 8.0 | 7.0 | 14.9 | 15.0 | 21.1 | 22.5 | 14.7 | 4.8 |
| Bunch filling time (days) | 98 | 94 | 96 | 97 | 102 | 108 | 116 | 127 | 129 | 108 | 119 | 97 |
| **ACO-*ZmPsy1* + Exp1-*PaCrtI*** | | | | | | | | | | | | |
| β-CE (µg/g DW) | 4.0 | 2.6 | 5.3 | 8.2 | N/A | 9.7 | 18.9 | 38.4 | 32.4 | 17.1 | 14.7 | 1.9 |
| Bunch filling time (days) | 117 | 90 | 92 | 98 | N/A | 108 | 122 | 116 | 118 | 126 | 108 | 99 |
| **Ubi-*MtPsy2a*** | | | | | | | | | | | | |
| β-CE (µg/g DW) | N/A | 18.8 | 8.8 | 16.6 | 28.1 | 37.1 | 17.2 | 60.6 | 46.3 | 46.9 | 3.2 | 8.4 |
| Bunch filling time (days) | 49 | 98 | 92 | 95 | 102 | 107 | 115 | 127 | 128 | 130 | 105 | 97 |
| **Ubi-*ZmPsy1*** | | | | | | | | | | | | |
| β-CE (µg/g DW) | N/A | 31.4 | 38.2 | 41.4 | 27.4 | 46.7 | 41.7 | 63.1 | 50.2 | 45.0 | 30.0 | 33.9 |
| Bunch filling time (days) | N/A | 119 | 97 | 102 | 101 | 83 | 122 | 129 | 137 | 118 | 125 | 109 |

**
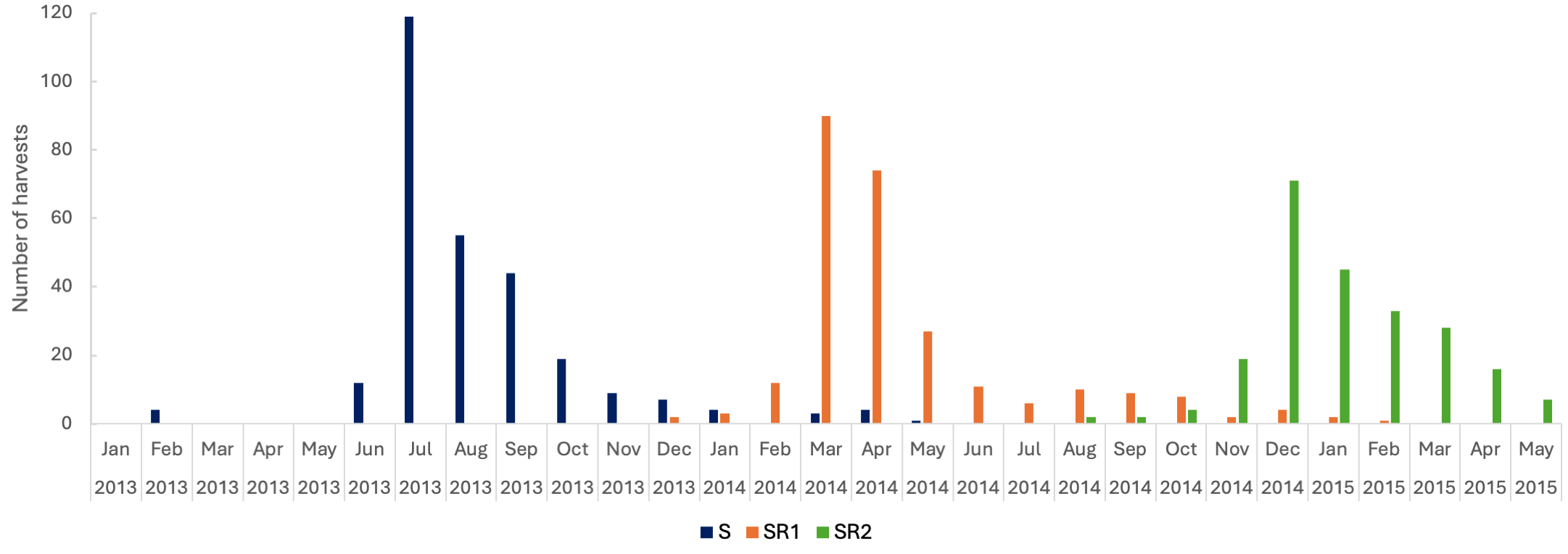
**

**Figure S6** Number of harvests for the sucker (S), sucker ratoon 1 (SR1) and sucker ratoon 2 (SR2) crops analysed in this study.

**Table S11.** Influence of environmental conditions on the accumulation of carotenoids in the green mature fruit of wild-type and transgenic ‘Cavendish’ banana calculated via two-tailed Pearson correlation

| **Line** | **Correlation** | **β-CE (µg/g DW) vs Bunch fill (days)** | **β-CE (µg/g DW) vs Temp (ᵒC)** | **β-CE (µg/g DW) vs Rainfall (mm)** | **β-CE (µg/g DW) vs Solar exposure (MJ/m2)** |
| --- | --- | --- | --- | --- | --- |
| WT | r | 0.7453 | -0.8683 | -0.5382 | -0.5119 |
|  | p-value | 0.0212 | 0.0024 | 0.1350 | 0.1589 |
| Exp1-*ZmPsy1* | r | 0.5052 | -0.8179 | -0.3958 | -0.5339 |
|  | p-value | 0.0939 | 0.0012 | 0.2028 | 0.0738 |
| ACO-*ZmPSY1* | r | 0.7373 | -0.2546 | -0.5935 | 0.2988 |
|  | p-value | 0.0062 | 0.4246 | 0.0419 | 0.3455 |
| ACO-*ZmPsy1*-Exp1-*PaCrtI* | r | 0.6180 | -0.6227 | -0.6489 | -0.0856 |
|  | p-value | 0.0427 | 0.0407 | 0.0308 | 0.8024 |
| Ubi-*ZmPsy1* | r | 0.2843 | -0.5868 | -0.4120 | -0.1133 |
|  | p-value | 0.3968 | 0.0577 | 0.2080 | 0.7400 |
| Exp1-*MtPsy2a* | r | 0.3219 | -0.7265 | -0.2471 | -0.6457 |
|  | p-value | 0.3644 | 0.0173 | 0.4912 | 0.0437 |
| ACO-*MtPsy2a* | r | 0.6313 | -0.3635 | -0.3167 | -0.0365 |
|  | p-value | 0.0503 | 0.3018 | 0.3726 | 0.9203 |
| Ubi-*MtPsy2a* | r | 0.8208 | -0.5456 | -0.4551 | -0.1011 |
|  | p-value | 0.0020 | 0.0826 | 0.1596 | 0.7675 |

|  | Moderate correlation |
| --- | --- |
|  | Strong correlation |
|  | Very strong correlation |

**Table S12** Influence of environmental conditions on bunch filling time in wild-type and transgenic ‘Cavendish’ banana calculated via two-tailed Spearman correlation

| **Line** | **Correlation** | **Bunch fill (days) vs Temp (ᵒC)** | **Bunch fill (days) vs Rainfall (mm)** | **Bunch fill (days) vs Solar exposure (MJ/m2)** |
| --- | --- | --- | --- | --- |
| WT | r | -0.3687 | -0.9289 | 0.2259 |
|  | p-value | 0.3236 | 0.0008 | 0.5582 |
| Exp1-*ZmPsy1* | r | -0.4311 | -0.9263 | 0.3404 |
|  | p-value | 0.1612 | <0.0001 | 0.2769 |
| ACO-*ZmPSY1* | r | -0.5866 | -0.8175 | 0.0772 |
|  | p-value | 0.0481 | 0.0018 | 0.8124 |
| ACO-*ZmPsy1*-Exp1-*PaCrtI* | r | -0.4828 | -0.6651 | 0.1276 |
|  | p-value | 0.1337 | 0.0293 | 0.7078 |
| Ubi-*ZmPsy1* | r | -0.0229 | -0.7091 | 0.5545 |
|  | p-value | 0.9512 | 0.0182 | 0.0818 |
| Exp1-*MtPsy2a* | r | -0.5016 | -0.8545 | 0.1273 |
|  | p-value | 0.1425 | 0.0029 | 0.7330 |
| ACO-*MtPsy2a* | r | -0.5675 | -0.9483 | -0.0243 |
|  | p-value | 0.0909 | 0.0001 | 0.9530 |
| Ubi-*MtPsy2a* | r | -0.5126 | -0.8273 | 0.1364 |
|  | p-value | 0.1097 | 0.0027 | 0.6937 |

|  | Moderate correlation |
| --- | --- |
|  | Strong correlation |
|  | Very strong correlation |

**Table S13** Correlations - Influence of green mature fruit carotenoid content on agronomical characteristics in wild-type and transgenic ‘Cavendish’ banana calculated via two-tailed Pearson correlation

| **Line** | **Correlation** | **β-CE (µg/g DW) vs Plant height (cm)** | **β-CE (µg/g DW) vs Plant cycle time (days)** | **β-CE (µg/g DW) vs Bunch cycle time (days)** | **β-CE (µg/g DW) vs Bunch weight (kg)** | **β-CE (µg/g DW) vs Finger number** | **β-CE (µg/g DW) vs Finger length (cm)** |
| --- | --- | --- | --- | --- | --- | --- | --- |
| WT | r | -0.6007 | 0.5041 | 0.7979 | -0.6694 | -0.7590 | -0.2602 |
|  | p-value | 0.0179 | 0.0554 | 0.0004 | 0.0063 | 0.0010 | 0.3490 |
| Exp1-*ZmPsy1* | r | -0.8530 | 0.5328 | 0.3824 | -0.8493 | -0.8341 | -0.7519 |
|  | p-value | <0.0001 | 0.0409 | 0.1596 | <0.0001 | 0.0001 | 0.0012 |
| ACO-*ZmPSY1* | r | -0.6288 | 0.6390 | 0.4473 | -0.7709 | -0.8627 | -0.1306 |
|  | p-value | 0.0285 | 0.0253 | 0.1448 | 0.0033 | 0.0003 | 0.6858 |
| ACO-*ZmPsy1*-Exp1-*PaCrtI* | r | 0.2108 | 0.2338 | 0.6783 | -0.7164 | -0.8551 | -0.1549 |
|  | p-value | 0.5861 | 0.5449 | 0.0446 | 0.0299 | 0.0033 | 0.6907 |
| Ubi-*ZmPsy1* | r | 0.4539 | -0.4290 | 0.4592 | 0.6755 | 0.3568 | 0.6741 |
|  | p-value | 0.4426 | 0.4710 | 0.4366 | 0.2108 | 0.5555 | 0.2121 |
| Exp1-*MtPsy2a* | r | -0.6211 | 0.0933 | 0.4481 | -0.4942 | -0.4316 | -0.4744 |
|  | p-value | 0.0311 | 0.7731 | 0.1440 | 0.1025 | 0.1613 | 0.1192 |
| ACO-*MtPsy2a* | r | -0.6566 | 0.0762 | 0.4385 | -0.7127 | -0.4019 | -0.6054 |
|  | p-value | 0.0078 | 0.7873 | 0.1020 | 0.0029 | 0.1375 | 0.0168 |
| Ubi-*MtPsy2a* | r | -0.5178 | 0.8504 | 0.6489 | -0.7810 | -0.6601 | -0.8073 |
|  | p-value | 0.0847 | 0.0005 | 0.0224 | 0.0027 | 0.0195 | 0.0015 |

|  | Moderate correlation |
| --- | --- |
|  | Strong correlation |
|  | Very strong correlation |

**Table S14** Carotenoid content in the leaves of selected wild-type and transgenic ‘Cavendish’ banana

| **Promoter-*transgene*** | **Line ID** | **Carotenoid content (µg/g DW)** | | | | | | |
| --- | --- | --- | --- | --- | --- | --- | --- | --- |
|  |  | **lutein** | **α-carotene** | ***trans* β-carotene** | ***cis* β-carotene** | **Total carotenoids** | **Total pVAC** | **β-CE** |
| Wild-type | FT162 | 1245.9±235.4 | 131.1±27.4 | 325.1±102.8 | 48.0±12.7 | 1750.2±338.7 | 504.2±103.5 | 438.7±108.7 |
|  | FT166 | 1362.0±316.2 | 122.9±46.3 | 342.4±88.7 | 50.5±10.8 | 1877.8±427.6 | 515.8±125.3 | 454.4±110.7 |
|  | FT167 | 1372.0±144.1 | 174.7±43.4 | 352.2±66.4 | 51.8±7.3 | 1950.7±207.6 | 578.7±65.0 | 491.4±65.9 |
|  | FT430 | 1289.5±430.8 | 133.4±85.2 | 355.0±117.1 | 51.1±16.5 | 1829.0±603.5 | 539.5±173.4 | 472.8±148.0 |
|  | FT448 | 1274.1±259.2 | 142.8±101.8 | 331.8±58.4 | 46.9±7.8 | 1795.5±390.5 | 521.4±132.5 | 450.0±91.3 |
| Exp1-*MtPsy2a* | FT242 | 1173.8±140.2 | 184.2±36.3 | 346.5±46.0 | 44.8±4.4 | 1749.4±214.2 | 575.6±77.2 | 483.4±62.4 |
|  | FT246 | 932.5±145.3 | 227.3±64.0 | 469.5±106.0 | 36.0±6.8 | 1665.3±230.9 | 732.8±148.1 | 619.2±124.0 |
|  | FT341 | 1070.2±240.1 | 152.4±45.3 | 282.1±76.6 | 37.8±8.0 | 1542.5±328.6 | 472.3±96.0 | 396.1±87.4 |
|  | FT342 | 1044.9±227.0 | 103.7±64.3 | 286.1±67.0 | 39.9±8.1 | 1474.6±345.0 | 429.7±119.3 | 377.8±93.8 |
| Exp1-*ZmPsy1* | FT187 | 1142.8±145.1 | 150.8±59.2 | 342.2±41.5 | 46.2±6.0 | 1682.0±203.3 | 539.2±79.1 | 463.8±57.7 |
|  | FT192 | 252.5±44.6 | 374.6±130.1 | 611.1±236.7 | 9.8±2.5 | 1248.1±383.7 | 995.6±367.5 | 808.3±302.6 |
|  | FT201 | 1058.9±235.7 | 229.9±72.9 | 510.9±133.5 | 41.9±5.3 | 1841.6±283.6 | 782.7±202.9 | 667.7±167.8 |
|  | FT538 | 1054.3±142.0 | 220.8±86.1 | 420.0±70.1 | 36.1±7.0 | 1731.2±131.1 | 676.9±135.5 | 566.5±100.3 |

Data represent means ± SD from 4 plants with 3 technical replicates (n=12) collected from the sucker crop. β-CE, β-carotene equivalents.

**Table S15** Carotenoid content in the peel of selected wild-type and transgenic ‘Cavendish’ banana

| **Promoter-*transgene*** | **Line ID** | **Carotenoid content (µg/g DW)** | | | | | | | |
| --- | --- | --- | --- | --- | --- | --- | --- | --- | --- |
|  |  | **lutein** | **α-carotene** | ***trans* β-carotene** | ***cis* β-carotene** | **Total carotenoids** | **Total pVAC** | **β-CE** |  |
| Wild-type | FT166 | 256±17 | 51.2±5.8 | 48.6±7.6 | 0±0 | 355.8±30.2 | 99.8±13.3 | 74.2±10.5 |  |
|  | FT430 | 230.2±44.7 | 150.6±25.4 | 51.3±3.6 | 0±0 | 432±73.3 | 201.8±28.6 | 126.5±16 |  |
|  | FT448 | 238.3±16.9 | 106.6±12.1 | 46.2±4.1 | 0±0 | 391.1±29.9 | 152.8±15.9 | 99.5±10 |  |
| ACO-*MtPsy2a* | FT504 | 257.9±39 | 101.9±15.6 | 56.8±7.9 | 0.6±1.2 | 417.1±47.2 | 159.2±8.5 | 108.3±1.8 |  |
|  | FT508 | 195.9±9.3 | 55.3±6.2 | 40.6±4 | 0±0 | 291.9±18.4 | 95.9±9.1 | 68.3±6.3 |  |
|  | FT511 | 309.4±22.7 | 78.7±4.7 | 64.8±2.5 | 1.5±2.9 | 454.5±27.4 | 145±6.4 | 105.7±4.7 |  |
|  | FT518 | 289.9±39 | 95.8±20.1 | 75.4±9.1 | 5.9±4.1 | 467±69.7 | 177.2±31.3 | 129.2±21.4 |  |
| ACO-*ZmPsy1* | FT467 | 137.8±12.9 | 380.3±27.5 | 543.3±63.2 | 8.6±0.8 | 1070.1±82.7 | 932.2±88.3 | 742.1±75.9 |  |
|  | FT475 | 119.7±17.3 | 557.6±21.2 | 821.9±77.1 | 11.5±1.2 | 1510.7±93 | 1391.1±97.9 | 1112.2±87.9 |  |
|  | FT479 | 107.7±39.1 | 347.3±142.4 | 559±210.6 | 8.2±5.6 | 1022.2±375.5 | 914.5±351 | 740.9±282.6 |  |
|  | FT483 | 139.4±18.5 | 561±67.9 | 856.6±104.3 | 18±2.1 | 1575±180.9 | 1435.6±169.4 | 1155.1±136.4 |  |

Data represent means ± SD from 4 plants with 3 technical replicates (n=12) collected from the sucker crop. β-CE, β-carotene equivalents.

**Table S16** Carotenoid content in the pulp during the development of wild-type and transgenic ‘Cavendish’ banana fruits

| **Promoter-*transgene*** | **Phenotype** | **Fruit stage** | **Carotenoid content (µg/g DW)** | | | | | | |
| --- | --- | --- | --- | --- | --- | --- | --- | --- | --- |
|  |  |  | **lutein** | **α-carotene** | ***trans* β-carotene** | ***cis* β-carotene** | **Total carotenoids** | **Total pVAC** | **β-CE** |
| Wild-type | Non-transgenic | S3 | 2.5±0.6 | 0.7±0.2 | 2.1±0.5 | 0.4±0.1 | 5.6±0.4 | 3.1±0.7 | 2.7±0.6 |
|  |  | S6 | 3.0±0.7 | 0.7±0.2 | 1.1±0.4 | 0.3±0.1 | 5.0±1.2 | 2.0±0.7 | 1.7±0.6 |
|  |  | S9 | 3.8±1.1 | 1.4±0.4 | 1.9±1.2 | 0.4±0.2 | 7.5±0.8 | 3.7±1.9 | 3.0±1.6 |
|  |  | S12 | 5.2±1.2 | 2.5±1.0 | 1.8±1.2 | 0.2±0.2 | 9.8±1.5 | 4.6±2.4 | 3.3±1.9 |
|  |  | S15 | 11.3±0.9 | 7.6±3.0 | 3.4±1.9 | 0.3±0.1 | 22.6±4.1 | 11.3±5.0 | 7.5±3.5 |
|  |  | FG | 10.2±3.5 | 4.9±4.2 | 1.7±1.8 | 0.2±0.2 | 17.0±9.2 | 6.8±6.1 | 4.4±4.0 |
| ACO-*ZmPsy1* | ‘Golden’ peel transgenic banana | S3 | 4.1±3.0 | 1.4±0.2 | 3.9±1.6 | 0.4±0.6 | 9.8±2.1 | 5.7±0.8 | 5.0±0.9 |
|  |  | S6 | 7.0±5.1 | 2.7±1.8 | 2.6±0.3 | 0.3±0.4 | 12.6±7.7 | 5.6±2.5 | 4.2±1.6 |
|  |  | S9 | 10.7±6.6 | 4.9±3.9 | 2.6±1.2 | 0.3±0.4 | 18.5±12.1 | 7.8±5.5 | 5.3±3.5 |
|  |  | S12 | 8.3±0.1 | 3.8±0.1 | 1.5±0.1 | 0.0±0 | 13.6±0.3 | 5.3±0.2 | 3.4±0.1 |
|  |  | FG | 22.4±3.5 | 9.6±3.2 | 2.3±1.0 | 0.2±0.1 | 34.4±7.7 | 12.1±4.2 | 7.3±2.7 |
| ACO-*MtPsy2a* | Green peel transgenic banana | S3 | 3.2±0.4 | 1.3±0.3 | 6.8±1.7 | 1.2±0.2 | 12.5±2.3 | 9.4±2.1 | 8.7±2.0 |
|  |  | S6 | 3.7±1.1 | 2.1±0.3 | 5.1±2.2 | 0.9±0.3 | 11.8±1.7 | 8.2±2.7 | 7.1±2.5 |
|  |  | S9 | 5.1±2.1 | 3.7±0.3 | 5.5±3.6 | 1.0±0.5 | 15.3±2.4 | 10.2±4.3 | 8.4±4.2 |
|  |  | S12 | 7.7±3.5 | 8.1±0.8 | 5.6±3.1 | 0.7±0.4 | 22.1±0.8 | 14.4±4.2 | 10.3±3.8 |
|  |  | S15 | 18.2±6.0 | 31.3±9.9 | 14.2±9.6 | 2.3±1.9 | 66.0±15.3 | 47.8±21.4 | 32.1±16.4 |
|  |  | FG | 17.2±12.8 | 18.2±6.4 | 4.8±2.9 | 0.5±0.3 | 40.6±17.8 | 23.4±8.4 | 14.4±5.5 |

Data represent means ± SD of 3 technical replicates from 3, 2 and 3 biological replicates from wild-type, ACO-*ZmPsy1* and ACO-*MtPsy2a* lines respectively and collected from the ratoon 2 crop (RC2).

pVAC, pro-vitamin A carotenoids. β-CE, β-carotene equivalents. S3, S6, S9, S12 and S15 represent 3, 6, 9, 12 and 15 weeks post-bunch emergence, respectively. FG, full green.

**Table S17** Carotenoid content in the peel during the development of wild-type and transgenic ‘Cavendish’ banana fruits

| **Promoter-*transgene*** | **Phenotype** | **Fruit stage** | **Carotenoid content (µg/g DW)** | | | | | | |
| --- | --- | --- | --- | --- | --- | --- | --- | --- | --- |
|  |  |  | **lutein** | **α-carotene** | ***trans* β-carotene** | ***cis* β-carotene** | **Total carotenoids** | **Total pVAC** | **β-CE** |
| Wild-type | Non-transgenic | S3 | 271.0±61.7 | 114.7±0.6 | 55.8±8.4 | 4.6±6.5 | 446.0±76.0 | 175.0±14.3 | 117.7±14.6 |
|  |  | S6 | 320.6±90.3 | 189.4±102.3 | 56.4±19.3 | 0.9±1.5 | 567.3±210.6 | 246.6±120.6 | 151.9±69.5 |
|  |  | S9 | 340.3±89.7 | 155.1±110.8 | 54.2±24.0 | 0.0±0.0 | 549.6±220.8 | 209.3±134.7 | 131.7±79.3 |
|  |  | S12 | 397.3±142.3 | 160.6±135.2 | 63.9±23.7 | 0.0±0.0 | 621.9±294.1 | 224.6±153.7 | 144.2±86.8 |
|  |  | S15 | 566.0±62.6 | 276.8±75.6 | 87.9±11.6 | 0.0±0.0 | 930.6±149.8 | 364.6±87.2 | 226.3±49.4 |
|  |  | FG | 594.0±49.3 | 276.8±39.5 | 82.0±14.8 | 0.0±0.0 | 952.8±97.3 | 358.9±54.1 | 220.4±34.4 |
| ACO-*ZmPsy1* | ‘Golden’ peel transgenic banana | S3 | 240.2±2.2 | 1541.4±51.2 | 2410.4±21.0 | 63.1±7.2 | 4255.1±77.2 | 4014.9±79.4 | 3244.2±53.9 |
|  |  | S6 | 260.8±1.1 | 1651.4±253.2 | 2355.6±334.4 | 59.6±12.5 | 4327.5±598.9 | 4066.7±600.0 | 3241.0±473.4 |
|  |  | S9 | 289.3±80.1 | 1479.6±184.3 | 1938.0±167.9 | 52.3±10.1 | 3759.2±442.4 | 3469.8±362.3 | 2730.1±270.2 |
|  |  | S12 | 385.3±16.2 | 1384.1±31.2 | 1588.9±50.3 | 40.2±4.6 | 3398.4±97.8 | 3013.1±84.8 | 2321.1±69.5 |
|  |  | FG | 269.7±14.1 | 1528.1±92.8 | 1912.6±130.0 | 44.3±12.5 | 3754.7±221.2 | 3485.0±235.3 | 2721.0±188.9 |
| ACO-*MtPsy2a* | Green peel transgenic banana | S3 | 420.3±217.1 | 480.6±520.4 | 92.6±49.9 | 3.8±1.82 | 997.3±789.2 | 577.0±572.1 | 336.7±311.9 |
|  |  | S6 | 483.9±268.8 | 505.2±528.1 | 80.5±42.9 | 0.0±0.0 | 1069.5±839.7 | 585.7±571.0 | 333.1±306.9 |
|  |  | S9 | 495.5±195.6 | 395.5±418.1 | 81.5±26.8 | 1.6±2.7 | 974.0±622.0 | 478.5±442.5 | 280.8±233.7 |
|  |  | S12 | 498.1±217.0 | 412.3±387.3 | 78.5±23.5 | 0.0±0.0 | 988.9±618.0 | 490.8±410.1 | 284.6±216.5 |
|  |  | S15 | 525.4±58.8 | 225.2±27.2 | 70.2±0.6 | 0.0±0.0 | 820.7±86.6 | 295.4±27.8 | 182.8±14.2 |
|  |  | FG | 638.1±14.2 | 278.1±22.1 | 102.1±25.7 | 0.0±0.0 | 1018.3±52.6 | 380.1±39.6 | 241.1±31.5 |

Data represent means ± SD of 3 technical replicates from 3, 2 and 3 biological replicates from wild-type, ACO-*ZmPsy1* and ACO-*MtPsy2a* lines respectively and collected from the ratoon 2 crop (RC2).

pVAC, pro-vitamin A carotenoids. β-CE, β-carotene equivalents. S3, S6, S9, S12 and S15 represent 3, 6, 9, 12 and 15 weeks post-bunch emergence, respectively. FG, full green.
